# The hand of *Homo naledi*

**DOI:** 10.1038/ncomms9431

**Published:** 2015-10-06

**Authors:** Tracy L. Kivell, Andrew S. Deane, Matthew W. Tocheri, Caley M. Orr, Peter Schmid, John Hawks, Lee R. Berger, Steven E. Churchill

**Affiliations:** 1 Animal Postcranial Evolution Lab, Skeletal Biology Research Centre, School of Anthropology and Conservation, University of Kent, Marlowe Building, Canterbury CT2 7NR, UK; 2 Department of Human Evolution, Max Planck Institute for Evolutionary Anthropology, Deutscher Platz 6, Leipzig 04103, Germany; 3 Evolutionary Studies Institute and Centre for Excellence in PalaeoSciences, University of the Witwatersrand, Private Bag 3, Wits 2050, South Africa; 4 Department of Anatomy and Neurobiology, College of Medicine, University of Kentucky, MN 224 UK Medical Centre, Lexington, Kentucky 40536-0098, USA; 5 Department of Anthropology, Lakehead University, Thunder Bay Ontario, Canada P7K 1L8; 6 Human Origins Program, Department of Anthropology, National Museum of Natural History, Smithsonian Institution, Washington DC 20560, USA; 7 Department of Cell and Developmental Biology, University of Colorado School of Medicine, Aurora, Colorado 80045, USA; 8 Anthropological Institute and Museum, University of Zuerich, Winterthurerstrasse 190, Zuerich CH-8057, Switzerland; 9 Department of Anthropology, University of Wisconsin-Madison, Madison, Wisconsin 53593, USA; 10 Department of Evolutionary Anthropology, Duke University, Box 90383, Durham, North Carolina 27708-9976, USA

## Abstract

A nearly complete right hand of an adult hominin was recovered from the Rising Star cave system, South Africa. Based on associated hominin material, the bones of this hand are attributed to *Homo naledi*. This hand reveals a long, robust thumb and derived wrist morphology that is shared with Neandertals and modern humans, and considered adaptive for intensified manual manipulation. However, the finger bones are longer and more curved than in most australopiths, indicating frequent use of the hand during life for strong grasping during locomotor climbing and suspension. These markedly curved digits in combination with an otherwise human-like wrist and palm indicate a significant degree of climbing, despite the derived nature of many aspects of the hand and other regions of the postcranial skeleton in *H. naledi*.

## Abstract (web summary)

It is unclear to what extent early hominins were adapted to arboreal climbing. Here, the authors show that the nearly complete hand of *H. naledi* from South Africa has markedly curved digits and otherwise human-like wrist and palm, which indicates the retention of a significant degree of climbing.

## Graphical abstract

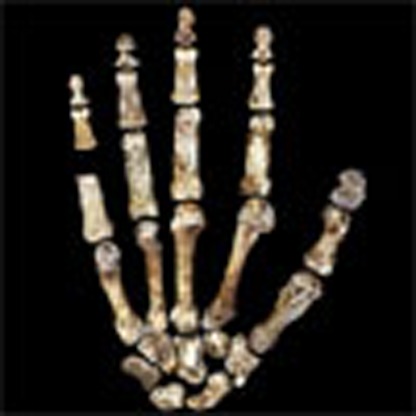

A longstanding palaeoanthropological debate concerns the degree to which arboreal climbing and suspension remained an important component of the early hominin behavioural repertoire. Hominin hand anatomy can provide valuable insights into this debate, but well-preserved hand bones are relatively rare in the fossil record and multiple hand bones from the same individual are even rarer. To date, nearly 150 hand bone specimens attributed to *H. naledi*^1^ have been uncovered from the Dinaledi Chamber of the Rising Star cave system^2^, representing at least six adults and two immature individuals. Twenty-six of these bones are from the right hand (Hand 1) of an adult individual. Missing only its pisiform (post mortem), this hand is part of the paratype of *H. naledi* and was recovered partially articulated with the palm up and fingers flexed (Fig. 1). This hand is small, similar in size to that of the *Australopithecus sediba* female MH2 (ref. 3), although there are other adult hand bones in the *H. naledi* sample that are slightly smaller and others slightly larger^1^. Here we focus on the comparative and functional morphology of this nearly complete hand.

Our comparative analyses reveal that the wrist and palm are generally most similar to those of Neandertals and modern humans, while the fingers are more curved than some australopiths. This distinctive mosaic of morphology has yet to be observed in any other hominin taxon and suggests the use of the hand for arboreal locomotion in combination with forceful precision manipulation typically used during tool-related behaviours.

## Results

### The thumb

Modern humans and archaic humans (as represented here by Neandertals) differ from other apes in having short fingers relative to a long and robust thumb with well-developed thenar musculature that facilitates forceful precision and precision-pinch grips between the thumb and fingers^4^,^5^,^6^. Most australopiths (for example, *Australopithecus afarensis* and *Australopithecus africanus*) have thumb-finger length proportions estimated to be similar to humans^7^,^8^,^9^ (but see ref. 10), but with gracile pollical metacarpals (Mc1) that lack strong muscle attachments^11^,^12^. The almost complete hand of *A. sediba* MH2 has a gracile but remarkably long thumb, outside the range of variation in recent humans^3^. Hand 1 also has a long thumb: the first ray length (Mc1+PP1=61.9 mm) is 58% of the third (Mc3+PP3+IP3=107.5 mm), falling only within the upper range of variation in modern human males (mean 55%) and outside the female range of variation (mean 54%; Fig. 2). The curvatures of the pollical carpometacarpal articulation fall within the modern human range of variation, unlike the more curved facets of extant great apes and some other early hominins^13^. Unlike most australopiths, Hand 1, as well as six additional Mc1 specimens from five other individuals, demonstrate that *H. naledi* has markedly robust pollical metacarpals with well-developed crests for the opponens pollicis and the first dorsal interosseous muscles (Fig. 3, Supplementary Fig. 1, Supplementary Table 1 and Supplementary Note 1). The former muscle is functionally important for opposition of the thumb to the fingers, as well as holding and manipulating large objects, whereas the latter muscle is strongly recruited during precision and precision-pinch grips^14^. In *H. naledi*, the flaring crests on the Mc1 for the intrinsic thenar muscles are accompanied by a prominent palmar ridge running sagittally along the midshaft (Fig. 3). Overall, the well-developed thenar muscle attachments are most similar to those seen in modern humans, Neandertals, and the Swartkrans pollical metacarpals (SK 84 and SKX 5020, attributed to either *A. (Paranthropus) robustus* or early *Homo*)^15^,^16^,^17^. In contrast, they are unlike the weakly developed muscle attachments of gracile australopiths^3^,^11^,^12^ and *Ardipithecus ramidus*^18^.

**Figure 1:**
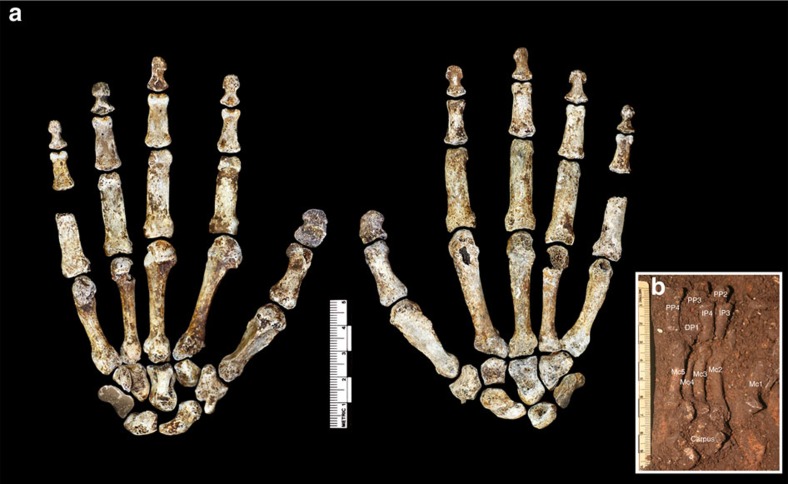
*H. naledi* Hand 1 adult right hand. (**a**) Palmar (left) and dorsal (right) views of the right hand bones, (**b**) found *in situ* in semi-articulation with the palm up and fingers flexed. The palmar surface of the metacarpals (Mc) and dorsal surface of the intermediate phalanges (IP) can be seen. DP, distal phalanx; PP, proximal phalanx.

**Figure 2:**
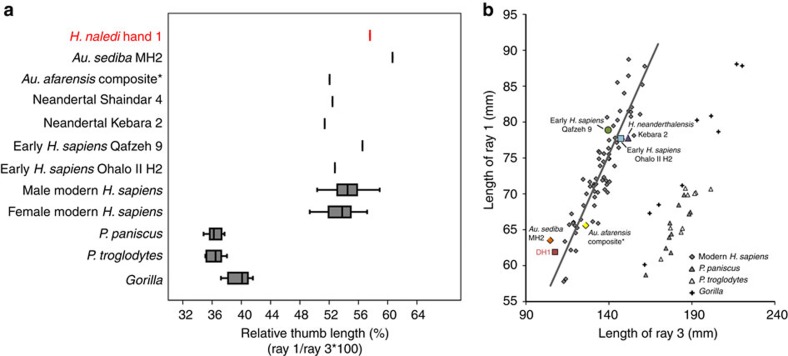
Relative length of the thumb in *H. naledi* Hand 1. Relative length of the thumb (ray 1, total length of the first metacarpal and first proximal phalanx) and third ray (total length of the third metacarpal and third proximal and intermediate phalanges) within the same individual, in all taxa except *A. afarensis* (*), for which the ratio is one potential estimate of hand proportions derived from multiple individuals^7^,^8^,^10^. (**a**) A box-and-whisker plot, where the box represents the 25th and 75th percentiles, the centre line represents the median and the whiskers represent the non-outlier range, of ray 1 to ray 3 length (as a percentage) demonstrates that Hand 1 has a relatively longer thumb than all other hominins, apart from *A. sediba*, and falls within the upper range of variation in modern human males only. (**b**) Linear regression of ray 1 length to ray 3 length, with regression line fit to modern humans (males and females combined), shows that Hand 1 (DH1) has a relatively long thumb for its small hand size, falling on the edge of modern human variation. Male and female modern human sample comprises African (*n*=31), Nubian Egyptian (*n*=11) and small-bodied Khoisan (*n*=25) individuals. Data for Shanidar 4 are derived from ref. 17.

**Figure 3:**
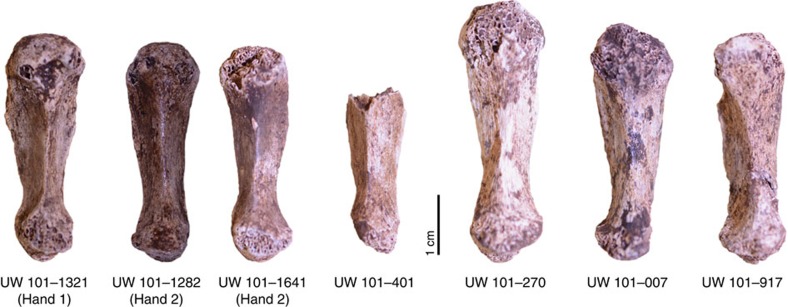
*H. naledi* pollical metacarpal (Mc1) morphology. Palmar view of the current sample of *H. naledi* Mc1s, including the right Mc1 of Hand 1, left and right Mc1 associated with a second individual (Hand 2) and four isolated Mc1s from four other individuals, demonstrating the homogeneity of *H. naledi* Mc1 morphology and variation in size across the sample.

Notwithstanding these similarities to modern humans and Neandertals, other aspects of the thumb morphology of *H. naledi* differ from these taxa in interesting ways. The base and proximal articular facet of the pollical metacarpal are remarkably small relative to its length, both radioulnarly and dorsopalmarly in Hand 1 and in the six additional pollical metacarpals from Dinaledi (Fig. 3 and Supplementary Fig. 1). The distal articular surface is also dorsopalmarly flat compared with other hominins and strongly asymmetric with a much larger palmar-radial protuberance. The *H. naledi* pollical distal phalanx (*n*=2) is large and robust; its apical tuft is radioulnarly broader relative to its length than those of australopiths, SKX 5016, Neandertals and modern humans (Fig. 1 and Supplementary Table 2). Its overall shape and apical tuft breadth most resemble the morphology of *Homo habilis* OH7 and *A. robustus* TM 1517k; however, unlike OH7, *H. naledi* demonstrates a well-developed ridge along the distal border of a deep proximal palmar fossa for the attachment of flexor pollicis longus tendon. The radial and ulnar tips of the apical tuft project proximopalmarly as ungual spines and there is a distinct area for the ungual fossa. Some of these features are found in early hominins^18^,^19^,^20^, but the full suite of features in *H. naledi* suggests it had a well-developed flexor pollicis longus muscle and a very broad, human-like palmar pad with a mobile proximal pulp^19^,^21^. These features facilitate forceful pad-to-pad gripping between the thumb and fingers^5^,^21^. The non-pollical distal phalanges corroborate this functional interpretation, being robust like that of Neandertals^17^ (There are two manual non-pollical distal phalanges from Dmanisi, Georgia^22^, associated with *Homo erectus*. However, although these phalanges are figured in ref. 22 and appear to also have broad apical tufts similar to modern humans, these fossils have not yet been described and no metrics are provided. Thus, formal comparisons with *H. erectus* distal phalanges are not yet possible)^22^ and more radioulnarly expanded than all australopiths and modern humans (Fig. 1 and Supplementary Table 2).

### The wrist

The robust pollical metacarpals of modern humans and Neandertals are associated with a suite of changes in carpal bone shape and articular configuration compared with extant great apes. These changes include a large Mc1 facet on the trapezium, a relatively large trapezium-scaphoid joint that extends onto the scaphoid tubercle, a boot-shaped trapezoid with an expanded palmar surface, a relatively large and more palmarly placed capitate-trapezoid articulation and the shift of a separate ossification centre from the capitate to the base of the Mc3 that results in a styloid process^5^,^13^,^23^,^24^. In addition, the Mc2 articulations with the trapezium and capitate are more proximodistally oriented, which acts to keep the trapezium-trapezoid and capitate-trapezoid joints in maximum contact during forceful precision and power grips^5^,^24^,^25^. Altogether, this derived complex of pollical and radial wrist features probably functions to distribute compressive loads and minimize shear during strong precision and precision-pinch grips involving the robust thumb and thenar musculature^13^,^24^,^26^.

Although some fossil hominins (for example, australopiths and OH7) variably share one or more of these features with modern humans and Neandertals, others (for example, *Homo floresiensis*) do not share any^11^,^27^,^28^,^29^,^30^,^31^,^32^. Overall, no early hominin taxon shows conclusive evidence that it had the full morphological complex or even a majority of the modern human features within it^13^,^31^,^32^,^33^. However, the absence of fossil wrist bones attributed to *H. erectus senso lato*, apart from a partial lunate^34^, complicates evolutionary interpretations of this anatomical region. The presence of a human-like styloid process on a Mc3 from Kaitio, Kenya (KNM-WT 51260), dated to ∼1.42 Ma and plausibly attributed to *H. erectus s.l.*, is the only evidence, suggesting that this complex may have arisen early in the evolution of the genus *Homo*^25^.

**Figure 4:**
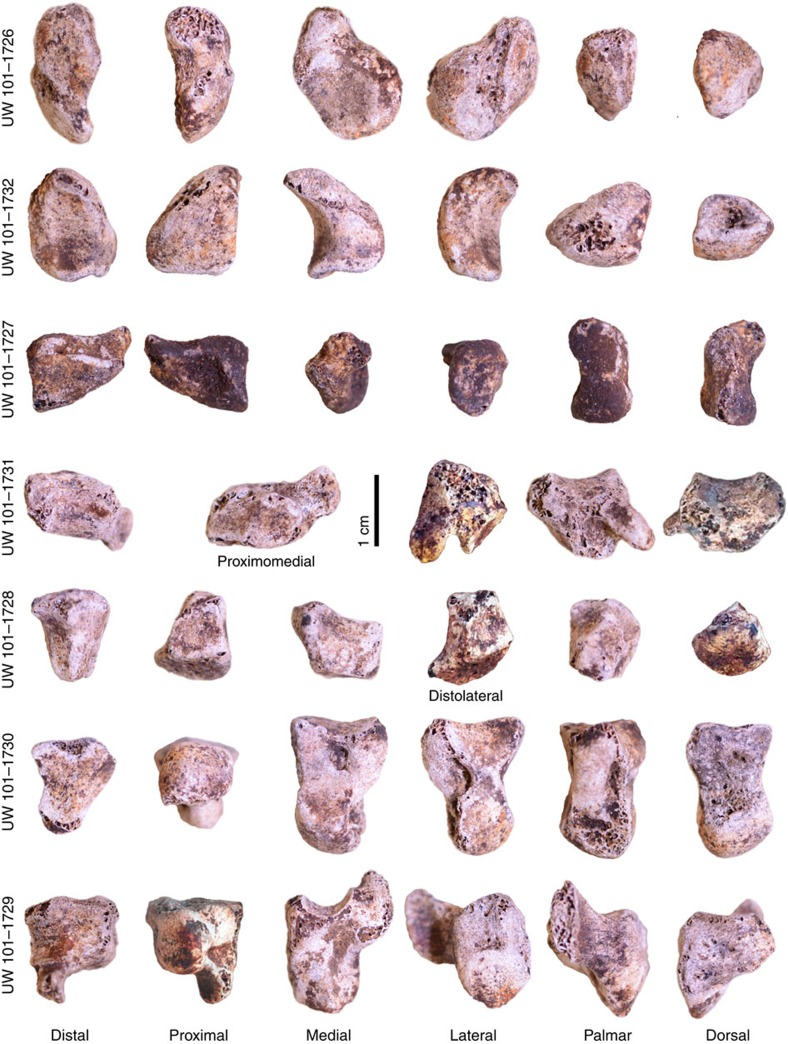
*H. naledi* Hand 1 wrist bones. The associated carpal bones of Hand 1, showing (from top to bottom) the scaphoid, lunate, triquetrum, trapezium, trapezoid, capitate and hamate in standard anatomical views. The trapezium is shown in proximomedial view to depict the trapezoid and scaphoid facets, and the trapezoid is shown in distolateral view to demonstrate the distinctive modern human-like ‘boot-shape’. All bones to scale.

**Figure 5:**
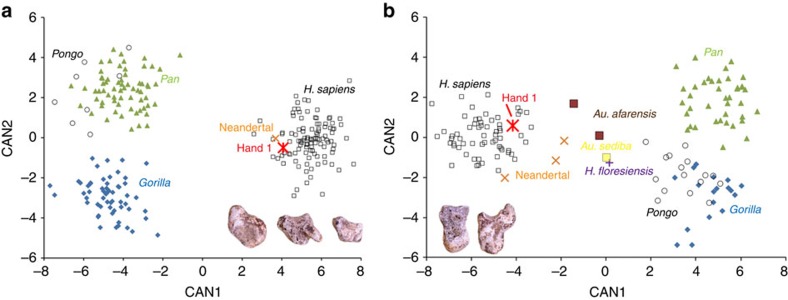
Three-dimensional multivariate analysis of *H. naledi* wrist bone shape. The first (CAN1) and second (CAN2) canonical variates of the (**a**) scaphoid, trapezium and trapezoid (STT, inset image) combined, including 15 angles, 13 relative areas and two curvatures, and (**b**) the capitate and hamate (CH, inset image) combined, including 12 angles, 9 relative areas and 4 other hamate metrics. In the STT analysis CAN1 and CAN2 explain 79.6% and 12.7% of the variance, respectively, and in the CH analysis CAN1 explains 84.8% and CAN2 8.9%, respectively. Fossil elements were analysed as test classification cases only and do not contribute to the observed variation along the canonical axes. In all cases, the posterior probabilities classify the Hand 1 (and Neandertal) wrist bones as 100% *H. sapiens*, compared with *A. afarensis* AL 333 classified as *H. sapiens* (50%) and *Pongo* (50%), *A. sediba* as *Gorilla* (52%) and *Pongo* (39%), and *H. floresiensis* as *Gorilla* (62%) and *Pongo* (36%).

In this context, the almost complete right wrist of Hand 1 provides a rare opportunity to examine this suite of carpal features in its entirety from a single fossil hominin individual (Figs 1 and 4). Comparative three-dimensional (3D) morphometric analyses of the scaphoid, trapezium and trapezoid (Fig. 5a and Supplementary Table 3a), and the capitate and hamate (Fig. 5b and Supplementary Table 3b) demonstrate that *H. naledi* wrist shape and articular configuration fall well within the ranges of variation seen in modern humans and Neandertals, and are thus derived relative to extant great apes, australopiths and *H. floresiensis*. Hand 1 and several other isolated carpal bones (Supplementary Table 1) have a relatively flat trapeziometacarpal joint, a facet for the trapezium that extends onto the scaphoid tubercle, an enlarged and palmarly expanded trapezoid–capitate joint and a boot-shaped trapezoid with an expanded palmar non-articular surface that probably repositions the thumb into a more supinated position compared with australopiths, OH7 and *H. floresiensis*^31^,^33^. Although the tubercle of the trapezium and the hamulus of the hamate are robust, both fall within the range of variation documented in modern humans and Neandertals (Fig. 4 and Supplementary Fig. 2).

However, some features in *H. naledi* fall near the edge or outside the ranges of variation observed in modern humans/Neandertals. Most striking are the small relative sizes of the trapezium’s Mc1 and scaphoid facets (12.3% and 6.6% of total trapezium area, respectively), which parallel the noticeably small Mc1 base and trapezial facet. (A single modern human outlier in our trapezium sample (*n*=119) displays a similar combination of even smaller relative articular areas of the Mc1 (10.9%) and scaphoid (5.3%) facets. Thus, although certainly atypical, relatively small facets such as those of the Hand 1 trapezium occasionally occur in modern humans.) The angle between the capitate’s second and third metacarpal facets of Hand 1 (108°) is also lower than that of any modern human (mean 140°±9°) or Neandertal (mean 132°±9°) in our sample (*n*=82) and is more similar to that seen in some australopiths and *H. floresiensis*^31^,^32^ (Supplementary Fig. 3). Finally, the *H. naledi* third metacarpal (*n*=3) lacks a styloid process, suggesting that the Mc3 styloid may not have arisen within the genus *Homo* as part of an evolutionarily integrated complex of radial carpometacarpal features. These specific morphological distinctions from the typical conditions observed in modern humans and Neandertals, however, do not detract from the otherwise overall similarity in carpal shape and articular configuration shared by Hand 1, modern humans and Neandertals (Figs 4 and 5). In conjunction with a robust and relatively long thumb, our results suggest that *H. naledi*, Neandertals and modern humans share a derived complex of features in the radial wrist and distal carpal row that distinguishes them from other early hominin taxa^24^,^31^,^32^,^33^.

### The non-pollical metacarpals

**Figure 6:**
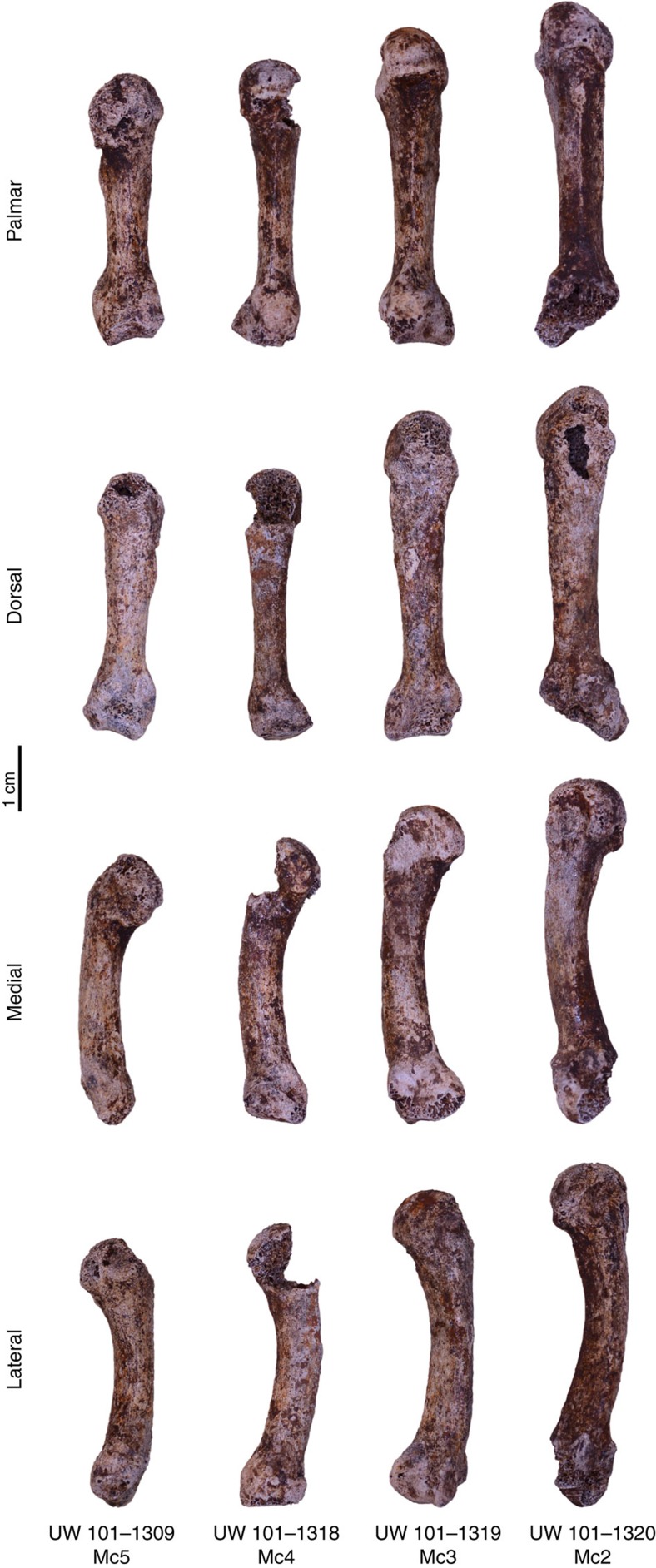
*H. naledi* Hand 1 non-pollical metacarpals. The associated medial metacarpals (Mc) of Hand 1 hand in standard anatomical views. Note the absence of the styloid process from the proximal base of the Mc3 and general robusticity of all the metacarpals, in particular the Mc5.

Modern human and Neandertal non-pollical metacarpals (Mc2–Mc5) differ from extant great apes in being relatively short and robust with asymmetrical head morphology (in particular that of the Mc2 and Mc5) and a saddle-shaped Mc5-hamate joint, all of which facilitate pad-to-pad contact between the fingers and thumb^5^,^17^,^33^,^35^. These features are absent in *A. ramidus*^18^, but most australopiths display derived, short, robust metacarpals with asymmetrical heads^3^,^7^,^11^,^12^. The Hand 1 metacarpals are generally similar in overall robusticity to most australopiths, Neandertals and modern humans, and do not have the unusually radioulnarly narrow metacarpal shafts typical of *A. sediba* MH2 (ref. 3) (Fig. 6 and Supplementary Figs 4–6). The *H. naledi* Mc5 (*n*=2) is particularly robust, such as that of *A. africanus* (StW 63) and Swartkrans SK(W) 14147, with a well-developed crest for the opponens digiti minimi muscle (Supplementary Fig. 6). However, unlike australopiths and the Swartkans specimens, *H. naledi* shares with modern humans a mildly saddle-shaped Mc5-hamate articulation, which facilitates rotation of the fifth digit towards the thumb and index finger^35^. Overall, *H. naledi*, Neandertals and moderns humans share metacarpal morphology that is consistent with an enhanced and derived ability to cup and manipulate objects within one hand relative to extant great apes^5^,^35^.

### The phalanges

**Figure 7:**
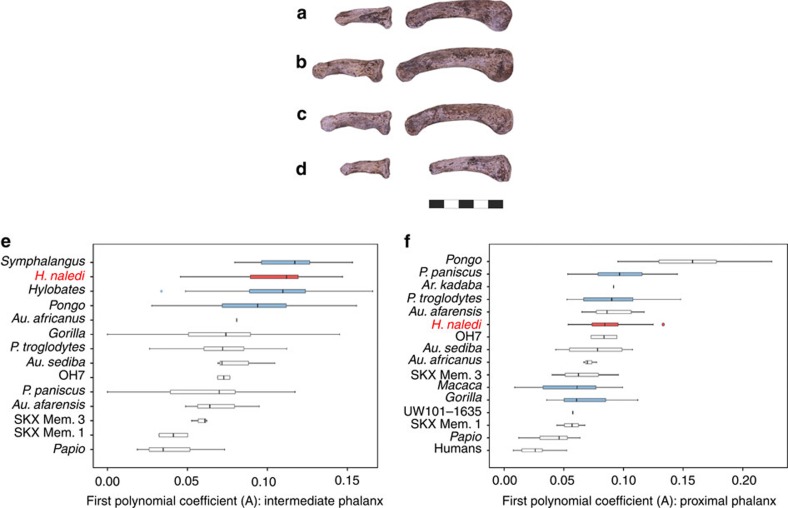
Phalangeal curvature in *H. naledi*. Above, proximal and IPs of the Hand 1 (**a**) second, (**b**) third (**c**) fourth and (**d**) fifth ray in lateral view (all to scale). Below, box-and-whisker plots of curvature in *H. naledi* (**e**) intermediate phalanges (*n*=14) and (**f**) proximal phalanges (*n*=11), quantified as the first polynomial coefficient (A) of the polynomial functions (*y*=A*x*^2^+B*x*+C) representing longitudinal shaft curvature of the dorsal surface. Vertical line represents the median value, boxes show the interquartile range and whiskers extend to the highest and lowest values of each taxon, excluding outliers (dots). The *H. naledi* sample is shown in red and extant taxa that are not statistically distinct from this sample (*P*≤0.05 based on one-way analysis of variance with Bonferroni correction) are shown in blue. ‘SKX Mem. 1’ and ‘SKX Mem. 3’ refer to the Swartkans phalanx sample from Members 1 and 3, respectively, that can be attributed to either *A. robustus* or early *Homo*. ‘UW 101-1635’ is a juvenile *H. naledi* proximal phalanx. *H. naledi* is unusual compared with most other hominins in having both strongly curved proximal and intermediate phalanges.

Modern human and Neandertal proximal and intermediate phalanges (IPs) are shorter, less curved and less robust, with poorly developed flexor tendon attachments compared with those of extant great apes (Fig. 7). Australopiths and OH7 generally demonstrate an intermediate condition, being slightly longer, more curved and/or more robust than the typical modern human/Neandertal morphology, but less so than observed in the extant apes^11^,^12^,^27^,^30^,^36^.

In comparison with the generally modern human/Neandertal-like morphology of the *H. naledi* wrist, thumb and palm, the fingers of Hand 1 are long and remarkably curved, similar to those of extant apes and early hominins (Fig. 7, Supplementary Fig. 7 and Supplementary Table 4). The mean curvature of *H. naledi* proximal phalanges (PPs, *n*=11) is almost identical to that of *A. afarensis* and OH7, and is not statistically distinct from African apes. The mean curvature of the IPs (*n*=14) is higher than that of any other hominin and not statistically distinct from Asian apes (Fig. 7). Although there is variation across fossil hominins, a combination of both highly curved PPs and IPs is unusual; extant apes and most fossil hominins, such as *A. afarensis* and OH7, generally have more strongly curved PPs and comparatively straight IPs. Experimental, behavioural and morphological evidence has demonstrated that phalangeal curvature is an adaptive response to the habitual stresses of locomotion, with more arboreal primates, especially those that often engage in suspension or climbing, having stronger longitudinal curvature compared with more terrestrial primates^36^,^37^,^38^,^39^,^40^,^41^,^42^. Biomechanically, curvature reduces the overall strain experienced by the phalanx during flexed-finger grasping postures, because a curved bone is more closely aligned with the joint reaction forces^40^,^42^. Thus, the strong degree of phalangeal curvature in *H. naledi* is a clear functional indication that its fingers experienced high loads during grasping required for climbing or suspensory locomotion. Furthermore, the degree of phalangeal curvature has been shown to respond to mechanical loading throughout ontogeny; primates that are more arboreal as juveniles than as adults show less curvature in adulthood^43^. The *H. naledi* sample includes one immature PP (UW 101-1635) and its curvature is less than that of the *H. naledi* adult mean, but within the low range of the adult variation (Fig. 7). This ontogenetic evidence suggests that *H. naledi* adults were using their hands for climbing during life just as much, if not more so, than the juveniles.

Hand 1 also has relatively long fingers. Relative finger-to-palm proportions vary strongly across fossil hominins and across different rays within individual hands (Supplementary Fig. 7). However, Hand 1 has longer third and fourth digits (PP length alone or PP+IP length/metacarpal length) than all other fossil hominins, except *A. ramidus* and the early modern human Qafzeh 9 (the latter with unusually long, but comparatively straight, phalanges). Hand 1 IPs are proportionately longer than those of australopiths and most later *Homo* individuals, and have a well-developed median bar indicative of high loading^44^. Long and curved fingers are consistent with the functional interpretation that *H. naledi* was using its hand for locomotor grasping. However, the phalangeal flexor sheath ridges on the PPs are not well-developed and are most similar in overall shape and robusticity to those of modern humans (Supplementary Fig. 8). Such morphology is consistent with the generally gracile morphology of the upper limb^1^ and may also be a biomechanical consequence of strong phalangeal curvature^42^. Overall, the remarkable curvature of both the PPs and IPs unambiguously indicates that locomotor grasping during climbing or suspension was a significant component of the *H. naledi* behavioural repertoire.

## Discussion

Over the course of human evolution, the hand was freed from the constraints of locomotion and has evolved primarily for manipulation. However, reconstructing the hands’ transition to bipedality and to tool use has been the source of much debate^5^,^6^,^33^,^35^,^45^. Furthermore, the few hand bones attributed to *H. erectus s.l*.^22^,^25^,^34^ are not an adequate sample from which to confidently test hypotheses about the evolution of the hominin wrist and hand during this transitional period. Australopiths and *H. habilis* are characterized by derived, human-like morphologies, primarily of the lower limb, which clearly indicate habitual bipedalism, but also varying suites of primitive, great ape-like features, primarily of the upper limb, which have elicited different functional interpretations. Some view the primitive features of early hominins as retentions from an arboreal ancestor that were either being lost or were selectively neutral and, as such, considered largely non-functional and adaptively insignificant^46^. Others, who aim to reconstruct early hominin behaviour as a whole, consider the primitive features as functionally useful with adaptive value retained under stabilizing selection^47^,^48^. Resolution of this debate requires morphological features that are ontogenetically sensitive to loading during life and, as such, can demonstrate how a bone was used during an individual’s lifetime^49^.

The strongly curved phalanges of *H. naledi* in association with an otherwise modern human/Neandertal-like hand, provide key evidence, consistent with primitive morphologies of the upper limb and thorax^1^, for the retention of a significant frequency of climbing in a fossil hominin biped^1^,^50^ that was also apparently adapted to the demands of intensified manipulative behaviours. The curvature of other early hominin (that is, australopiths and OH7) phalanges is intermediate between that of extant apes and modern humans, and it has been argued that these curved digits indicate frequent use of arboreal substrates in these hominins^47^,^48^. In contrast to the phalangeal morphology, the full suite of derived thumb and wrist features in Hand 1 is found only in committed, habitual tool users (for example, Neandertals and modern humans), suggesting that much of the hand anatomy in *H. naledi* may be the result of selection for precision handling and better distribution of compressive loads during forceful manipulative behaviours such as tool making and tool use (although tools have not been recovered in the Dinaledi Chamber itself^2^). Nevertheless, long and curved phalanges clearly suggest the use of the hand during life for powerful locomotor grasping and the functional importance of climbing in *H. naledi*. Therefore, as a whole, Hand 1 demonstrates that the ability for forceful precision manipulation is compatible with the use of the hand for arboreal locomotion. Whether or not this dual role required functional trade-offs that compromised the performance of these behaviours to some degree is currently unclear. When further considered within the context of the human-like foot^50^ and long lower limb^1^ in *H. naledi*, the hand morphology is consistent with the hypothesis that early hominins retained primitive use of the upper limb, even while fine-tuning specific aspects of the postcranial anatomy to facilitate novel behaviours such as efficient terrestrial locomotion and tool use.

## Methods

### Comparative morphometric analysis

*H. naledi* hand remains were compared with the morphology of the original fossils of *A. ramidus*, *A. afarensis*, *A. africanus*, *A. sediba*, *A. robustus*/early *Homo* from Swartkrans, *H. habilis*, *Homo neanderthalensis* and early modern *Homo sapiens*. Metric data were also compared with published data on *H. neanderthalensis* from Shanidar^17^, Krapina and Kiik-Koba^51^. The composite hand of *A. afarensis*, used to determine the estimated relative thumb length, includes the following specimens: AL333w-39 (Mc1), AL333-69 (PP1), AL333-16 (Mc3), AL333-63 (PP3) and AL333-88 (IP3), following Marzke^7^. The extant comparative sample included *Pan troglodytes*, *Pan paniscus*, *Gorilla gorilla*, *Gorilla berengei* and modern humans. The modern human sample comprised Nubian Egyptians, Europeans, Africans, small-bodied Khoisan and skeletally robust Tierra del Fuegians. Samples of extant taxa and specimens for fossil taxa varied depending on the analyses; thus, this information is provided for each analysis in the Supplementary Information.

Standard length and breadth measures of the metacarpals and phalanges were compared across the extant and fossil samples using box-and-whisker plots (Supplementary Figs 1 and 4–8). Metacarpal measurements included total and interarticular proximodistal length of the bone, maximum radioulnar breadth of the proximal and distal articular surfaces, and maximum radioulnar breadth of the proximal, mid- and distal shaft. Phalangeal measurements included total length and maximum radioulnar breadth of the proximal end, midshaft and trochlea.

### Multivariate analyses of 3D models

The 3D surface models of carpal bones were generated using laser and computed tomography scanning, following procedures outlined elsewhere (see Tocheri *et al.*^52^ and references therein). The articular and non-articular areas of each carpal bone were segmented using Geomagic Studio software, typically while using the actual bone as a reference. Scale-free metrics that capture different aspects of carpal shape were then quantified^53^,^54^,^55^. These metrics include curvatures (radioulnar and dorsopalmar) of the first metacarpal facet on the trapezium, relative areas of articular and non-articular surfaces, and angles between articular surfaces (see refs 13, 24, 56, 57 for further methodological details on these methods). These data were analysed using canonical variates and linear discriminant function analyses, to examine the overall morphometric similarities and differences among extant and fossil hominid taxa (Fig. 5). In the scaphoid–trapezium–trapezoid analysis (Fig. 5a), sample sizes were as follows: 106 modern humans, 65 *Pan*, 57 *Gorilla*, 8 *Pongo*, 1 Neandertal and 1 *H. naledi*; in the capitate–hamate analysis (Fig. 5b), sample sizes were as follows: 62 modern humans, 40 *Pan*, 18 *Gorilla*, 16 *Pongo*, 3 Neandertals, 1 *H. floresiensis*, 1 *A. sediba*, 2 *A. afarensis* and 1 *H. naledi*.

### High-resolution polynomial curve fitting methodology

All phalangeal curvatures were quantified using high-resolution polynomial curve fitting (HR-PCF) methods^36^,^41^,^58^. Although HR-PCF analysis is not software dependent, all curvatures were quantified using proprietary HR-PCF curve fitting software^41^. Unlike traditional curvature quantification techniques (that is, included angle and normalized curvature moment arm) that model curvature as an imaginary line passing through the centre of a bone, HR-PCF models the surface curvature of the bone and can fit a polynomial function to either the dorsal or palmar surface of a phalanx. The palmar surfaces of many phalanges were interrupted by flexor sheath ridges that create irregularities in the outline of shaft curvature; hence, the more regular dorsal margin of the outline was chosen for polynomial fitting. Although it could be argued that the dorsal and palmar curvatures are responses to different loading regimes, they are highly interdependent and associated with the same positional behaviour.

Elements were photographed in a lateral and standardized orientation. JASC PSP image-editing software was used to convert the resulting two-dimensional images into simple digitized outlines. These digitized outlines contain thousands of individual pixels, each having its own paired co-ordinates. End points were selected for each dorsal contour to represent the limits of a discrete second-order curve and the co-ordinates of the individual pixels comprising the selected portion of the dorsal contour were used as data points to generate a best-fit second-order polynomial function with three coefficients defined as *y=*A*x*2*+*B*x+*C. The three resulting co-efficients (A,B,C) can be used as the raw data in a statistical analysis. The first coefficient (A) expresses the nature and degree of the longitudinal curvature, whereas the second (B) and third (C) reflect aspects of the orientation of that curve with respect to the rest of the element (that is, element rotation and element position in two-dimensional space). Given the limitations of coefficients B and C to represent meaningful information about the magnitude of phalangeal shaft curvature, only the first (A) polynomial coefficient was considered in statistical analyses performed in the present study.

Although any order of polynomial can be used with HR-PCF methods, a second-order polynomial was chosen over a higher-order polynomial functions, because second-order curves (for example, longitudinal phalangeal shaft curvature) have no structural points of inflection unlike third-order curves and above, which impose either one or more points of inflection. The coefficients of higher-order polynomials (that is, third to sixth order) are very sensitive to whatever irregularities exist in the contours of anatomical curves. A more detailed treatment of the HR-PCF method is presented in Deane *et al.*^41^.

The mean curvatures for discrete taxonomic samples were compared directly using a one-way analysis of variance with a Bonferroni correction.

## Additional information

**How to cite this article:** Kivell, T. L. *et al.* The hand of *Homo naledi*. *Nat. Commun.* 6:8431 doi: 10.1038/ncomms9431 (2015).

## Supplementary Material

Supplementary Information Supplementary Figures 1-8, Supplementary Tables 1-4, Supplementary Note 1 and Supplementary References
