# Supplementary material for: The hand of Homo naledi
Source: Nat Commun. 2015 Oct 6;6:8431. doi: 10.1038/ncomms9431 (PMC4597335; doi:10.1038/ncomms9431)
Supplement: Supplementary Information — Supplementary Figures 1-8, Supplementary Tables 1-4, Supplementary Note 1 and Supplementary References [file ncomms9431-s1.pdf]

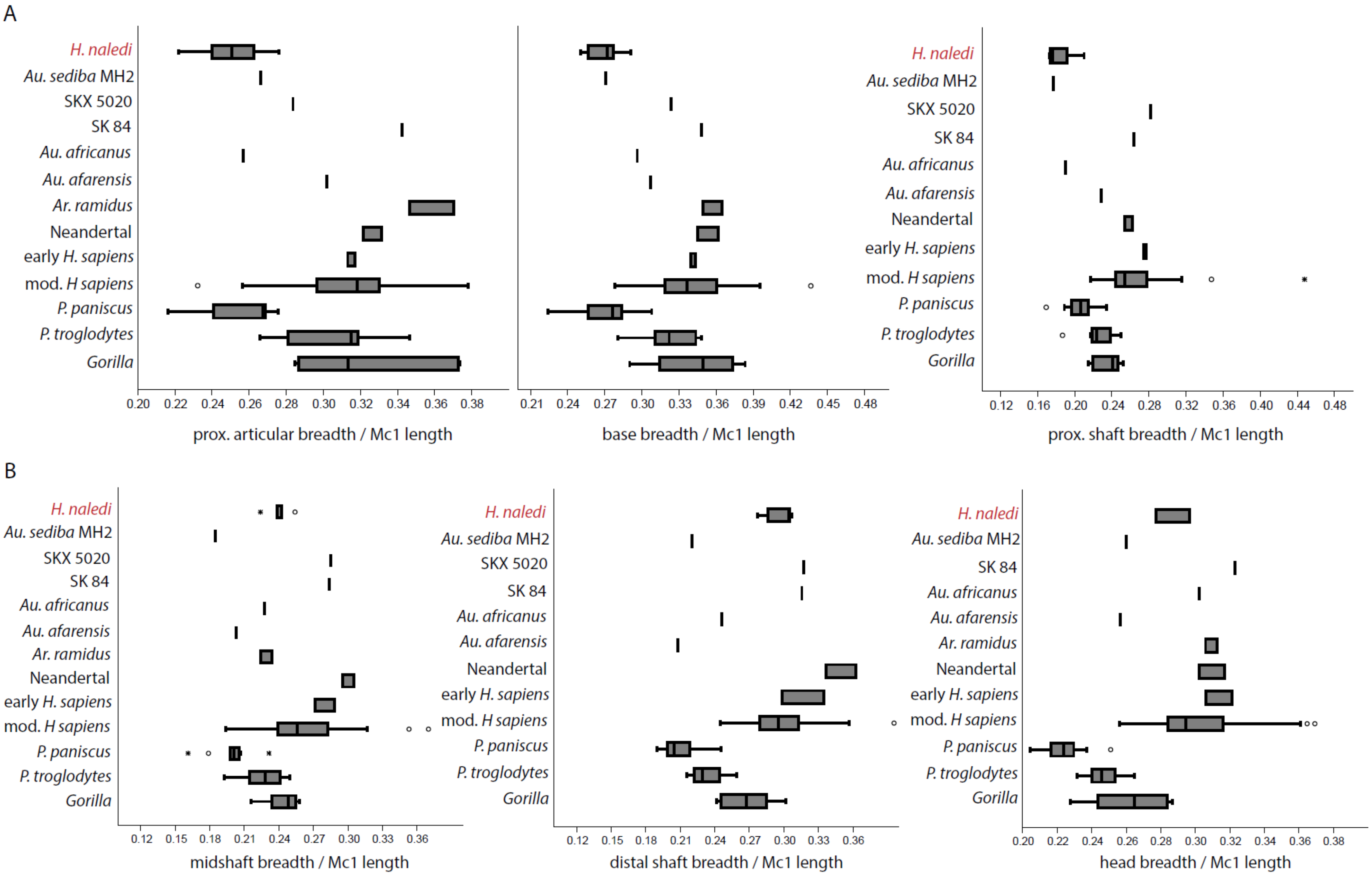
A
H. naledi
Au. sediba MH2
SKX 5020
SK 84
Au. africanus
Au. afarensis
Ar. ramidus
Neandertal
early H. sapiens
mod. H sapiens
P. paniscus
P. troglodytes
Gorilla
0.20 0.22 0.24 0.26 0.28 0.30 0.32 0.34 0.36 0.38
prox. articular breadth / Mc1 length
0.21 0.24 0.27 0.30 0.33 0.36 0.39 0.42 0.45 0.48
base breadth / Mc1 length
0.12 0.16 0.20 0.24 0.28 0.32 0.36 0.40 0.44 0.48
prox. shaft breadth / Mc1 length
B
0.12 0.15 0.18 0.21 0.24 0.27 0.30 0.33 0.36
midshaft breadth / Mc1 length
0.12 0.15 0.18 0.21 0.24 0.27 0.30 0.33 0.36
distal shaft breadth / Mc1 length
0.20 0.22 0.24 0.26 0.28 0.30 0.32 0.34 0.36 0.38
head breadth / Mc1 length

Supplementary Figure 1. Relative robusticity of the *H. naledi* first metacarpal (Mc1). Box-and-whisker plots depicting ratio of Mc1 total length and the maximum mediolateral breadth of (A) the proximal articular facet for trapezium, base and proximal shaft and (B) midshaft, distal shaft and distal head (below). Fossil samples include *H. naledi* UW 101-1321 from Hand 1, as well as UW 101-270, -007, -917, -1641 and -1282; *Au. sediba* MH2; *Au. africanus* StW 418; Swartkrans specimens SKX 5020 and SK 84 associated with *Au. robustus* or early *Homo*; *Au. afarensis* AL 333w-39; *Ar. ramidus* ARA-VP-6/500-015 and ARA-VP-6/1638; *H. neanderthalensis*, Kebara 2 and Amud 1; early *H. sapiens* Qafzeh 9 and Ohalo II H2 (mean of left and right Mc1s in the latter). All data were collected from original fossils. Modern human sample (n=68) ranges from small-bodied Khoisan to skeletally-robust Tierra del Fuegians. African ape sample includes *Pan paniscus* (n=11), *P. troglodytes* (n=10) and *Gorilla* (n=9).

H. naledi is unusual in having a remarkably mediolaterally narrow proximal end and articular facet, but a robust distal portion compared with other hominins. Relative dorsopalmar height of the same regions relative to Mc1 length shows a similar pattern.

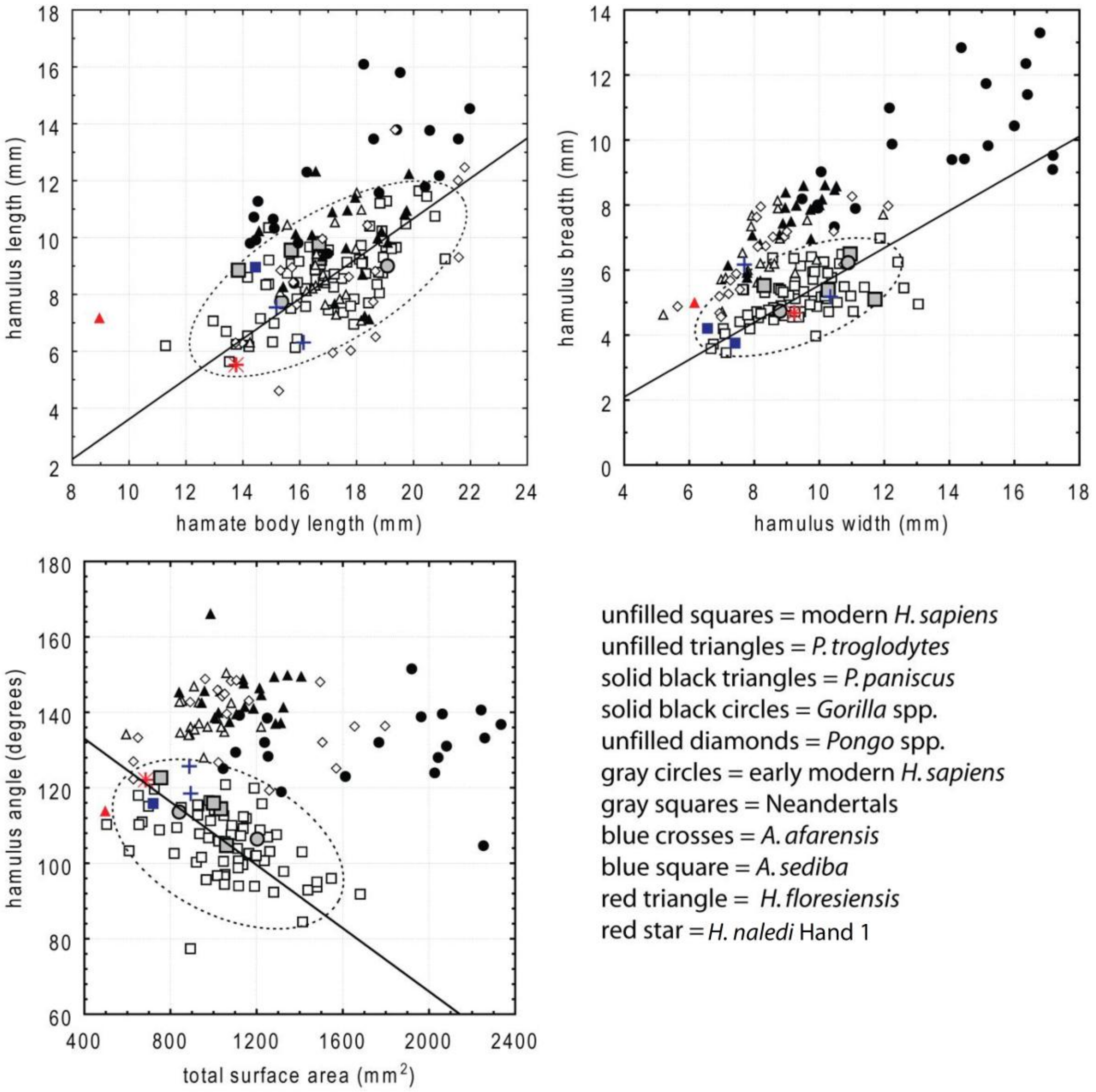

Supplementary Figure 2. Comparative analysis of the *H. naledi* hamate morphology. Depicted are bivariate plots of hamulus length relative to hamate body length (top left), hamulus breadth relative to hamulus width (top right) and angle of the hamulus relative to total surface area of the hamate (bottom left). Hamate measurements are described in Orr et al. (Ref S1). The Hand 1 hamulus morphology falls within in the range of variation observed in modern humans, but on the edge of variation for relative length and angle of the hamulus. There is a negative correlation between the angle of the hamulus and overall hamate size. Thus, although the *H. naledi* hamulus is somewhat distally inclined, it falls where expected for the small-bodied modern humans in this regard.

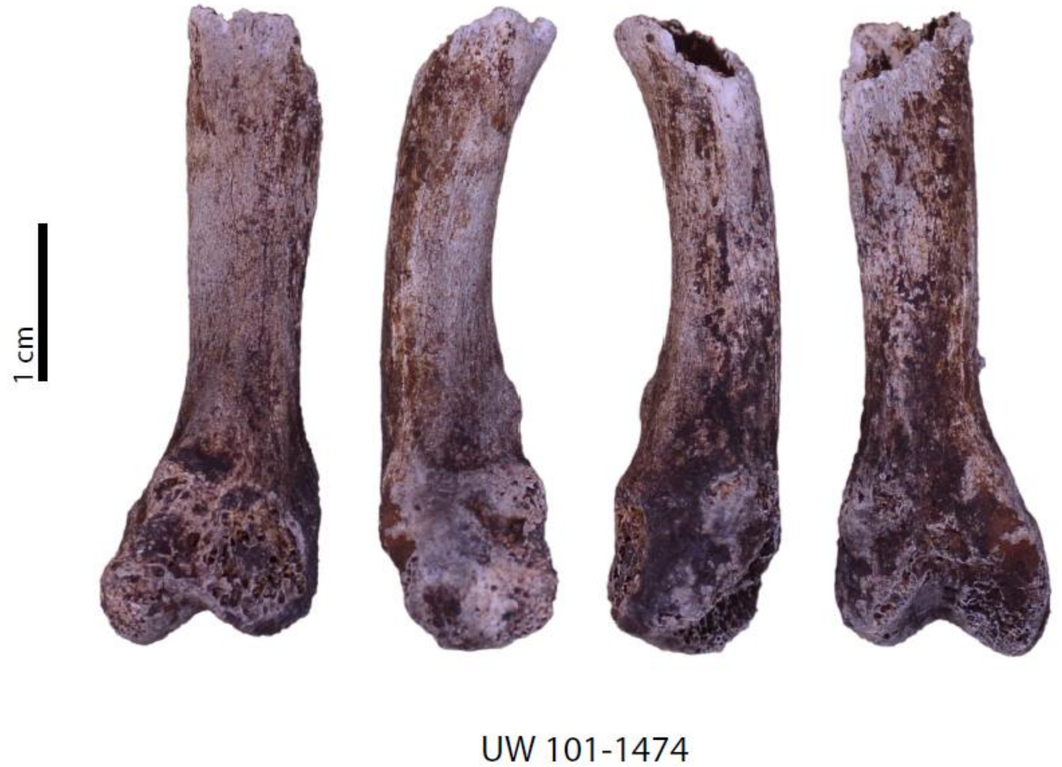

Supplementary Figure 3. *H. naledi* second metacarpal UW 101-1474 that better preserves the morphology of the proximal base than the Mc2 of Hand 1, shown in (from left to right) palmar, medial, lateral and dorsal views.

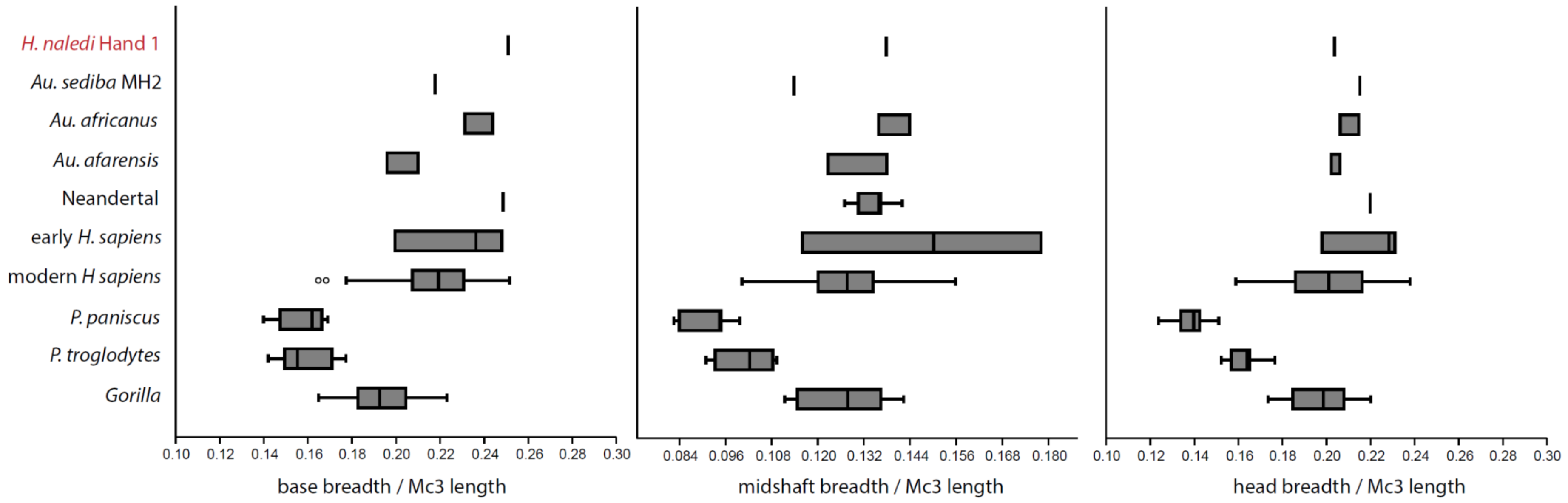

Supplementary Figure 4. Relative robusticity of the *H. naledi* Hand 1 third metacarpal (Mc3). Box-and-whisker plots depicting the ratio of interarticular length of the Mc3 and the maximum mediolateral breadth of the proximal base (left), midshaft (middle), and distal head (right). Fossil samples include *H. naledi* Hand 1 UW 101-1319; *Au. sediba* MH2; *Au. africanus* StW 68 and StW 64; *Au. afarensis* AL 438-1d and AL333-16; *H. neanderthalensis* La Ferrassie 1 and 2 (Ref S2), Regourdou 1 (Ref S2), Shanidar 4 and 6 (Ref S3) and Kebara 2; early *H. sapiens* Qafzeh 8, Qafzeh 9 and Ohalo II H2 (mean of left and right Mc3s in the latter). All data collected from original fossil unless otherwise indicated. Modern human sample (n=67) ranges from small-bodied Khoisan to skeletally-robust Tierra del Fuegians. African ape sample includes *Pan paniscus* (n=12), *P. troglodytes* (n=12) and *Gorilla* (n=11). Note that Mc3 length measure does not include the styloid process if present.

H. naledi Hand 1 Mc3 robusticity is generally similar to other hominins (apart from *Au. sediba*) and modern humans.

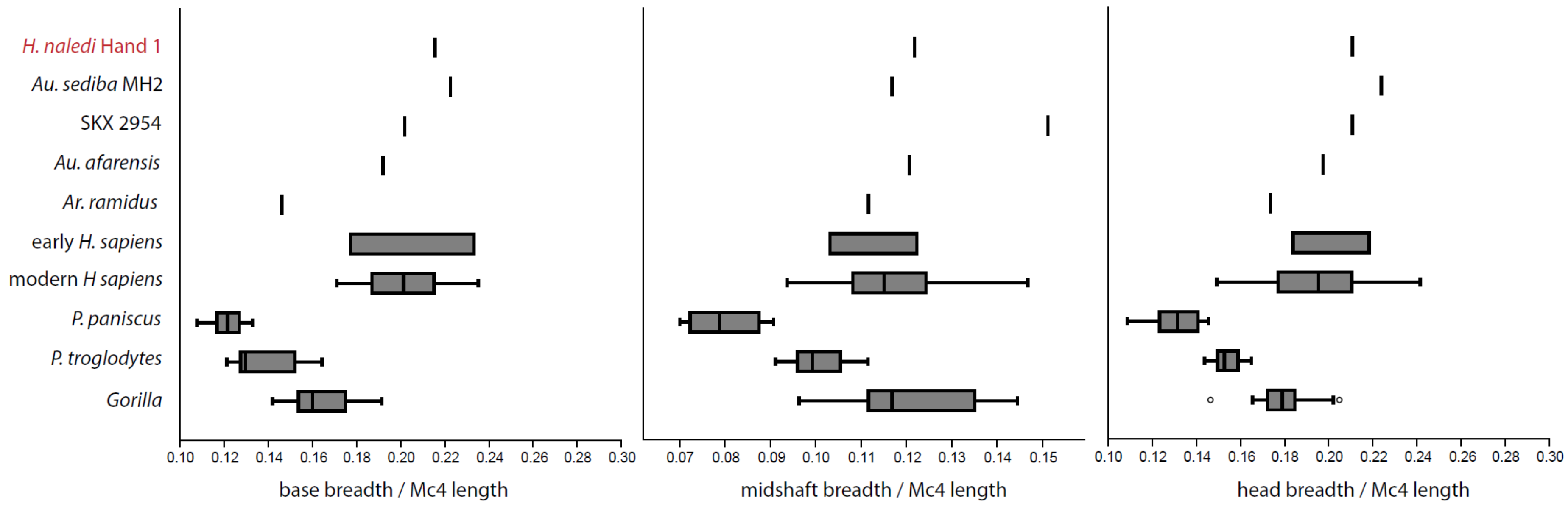

Supplementary Figure 5. Relative robusticity of the *H. naledi* Hand 1 fourth metacarpal (Mc4). Box-and-whisker plots depicting ratio of total length of the Mc4 and maximum mediolateral breadth of the proximal base (left), midshaft (middle), and distal head (right). Fossil samples include *H. naledi* Hand 1 UW 101-1318; *Au. sediba* MH2; SKX 2954 from Swartkrans associated with *Au. robustus* or early *Homo*; *Au. afarensis* AL333-56; *Ar. ramidus* ARA-VP-7/2G; early *H. sapiens* Qafzeh 9, Ohalo II H2 (mean of left and right Mc4s in the latter). All data collected from original fossils. Modern human sample (n=65) ranges from small-bodied Khoisan to skeletally-robust Tierra del Fuegians. African ape sample includes *Pan paniscus* (n=11), *P. troglodytes* (n=12) and *Gorilla* (n=11).

H. naledi Hand 1 Mc4 robusticity is generally similar to other australopiths and *H. sapiens* and unlike the more gracile *Ar. ramidus* and extant African apes.

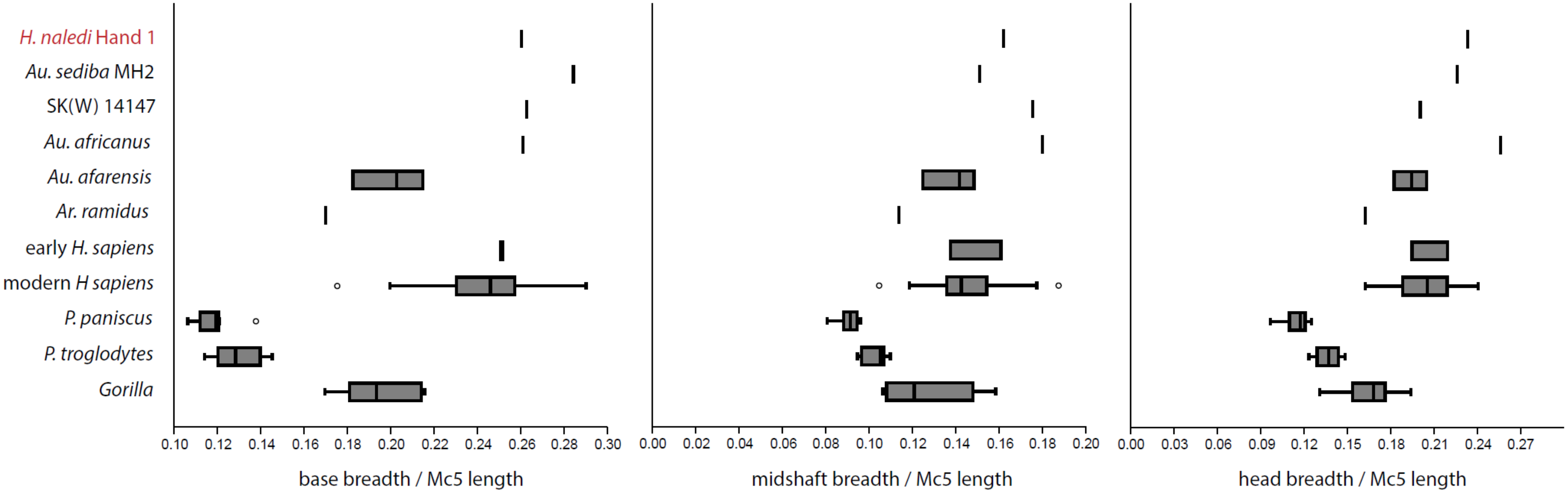

Supplementary Figure 6. Relative robusticity of the *H. naledi* Hand 1 fifth metacarpal (Mc5). Box-and-whisker plots depicting the ratio of total length of the Mc5 and maximum mediolateral breadth of the proximal base (left), midshaft (middle), and distal head (right). Fossil samples include *H. naledi* Hand 1 UW 101-1309; *Au. sediba* MH2; SK(W) 14147 from Swartkrans associated with *Au. robustus* or early *Homo*; *Au. africanus* StW 63; *Au. afarensis* AL333-14, AL333-89 and AL333-141; *Ar. ramidus* ARA-VP-6/500-036; early *H. sapiens* Qafzeh 9 and Ohalo II H2 (mean of left and right Mc5s in the latter). All data collected from original fossils. Modern human sample (n=62) ranges from small-bodied Khoisan to skeletally-robust Tierra del Fuegians. African ape sample includes *Pan paniscus* (n=11), *P. troglodytes* (n=11) and *Gorilla* (n=9).

H. naledi Hand 1 Mc5 is generally more robust than *Ar. ramidus, Au. afarensis* and extant African apes, and is most similar to other australopiths and *H. sapiens*.

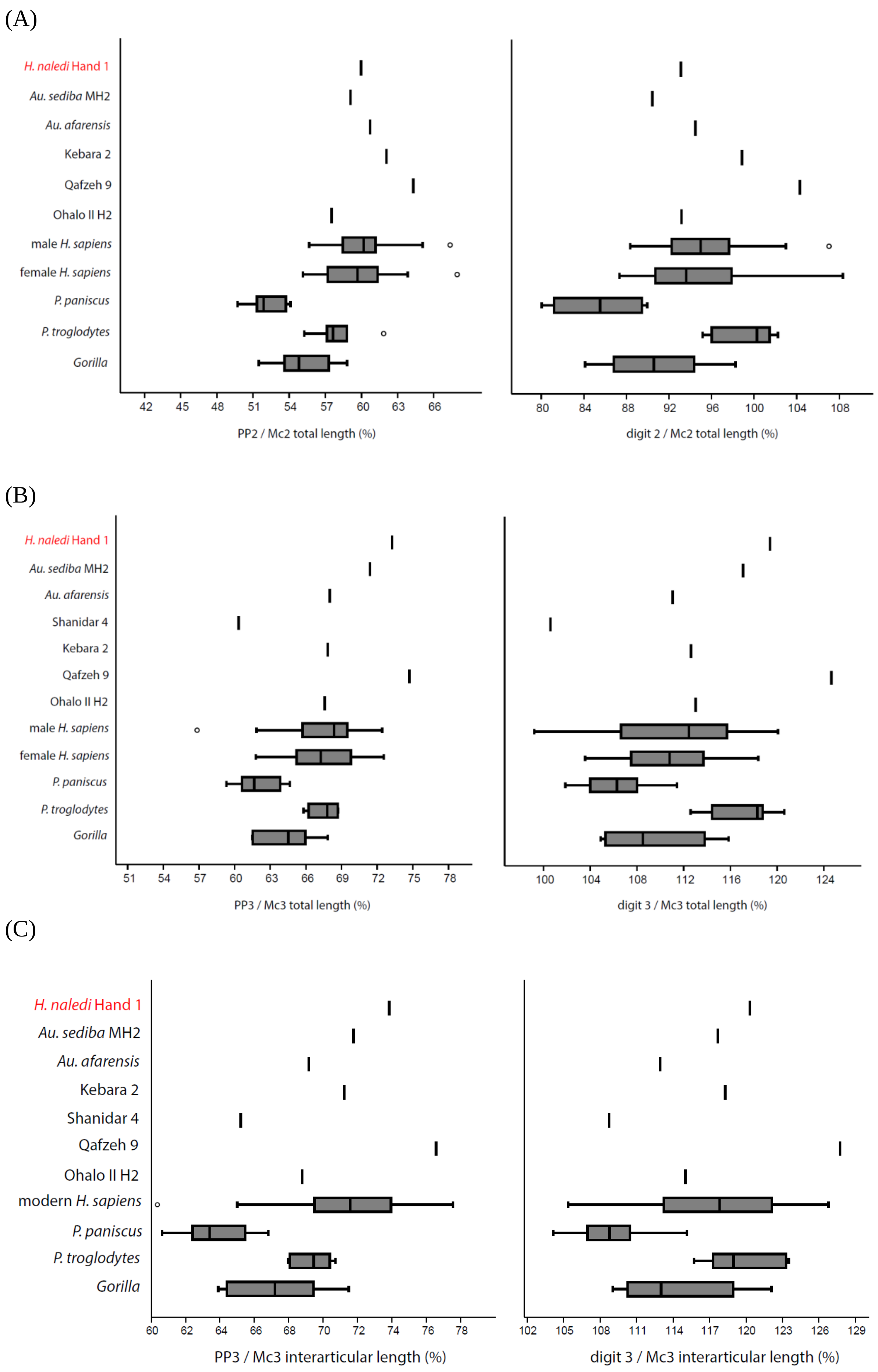
(A)
H. naledi Hand 1
Au. sediba MH2
Au. afarensis
Kebara 2
Qafzeh 9
Ohalo II H2
male H. sapiens
female H. sapiens
P. paniscus
P. troglodytes
Gorilla
42 45 48 51 54 57 60 63 66
PP2 / Mc2 total length (%)
80 84 88 92 96 100 104 108
digit 2 / Mc2 total length (%)
(B)
H. naledi Hand 1
Au. sediba MH2
Au. afarensis
Shanidar 4
Kebara 2
Qafzeh 9
Ohalo II H2
male H. sapiens
female H. sapiens
P. paniscus
P. troglodytes
Gorilla
51 54 57 60 63 66 69 72 75 78
PP3 / Mc3 total length (%)
100 104 108 112 116 120 124
digit 3 / Mc3 total length (%)
(C)
H. naledi Hand 1
Au. sediba MH2
Au. afarensis
Kebara 2
Shanidar 4
Qafzeh 9
Ohalo II H2
modern H. sapiens
P. paniscus
P. troglodytes
Gorilla
60 62 64 66 68 70 72 74 76 78
PP3 / Mc3 interarticular length (%)
102 105 108 111 114 117 120 123 126 129
digit 3 / Mc3 interarticular length (%)

(D)

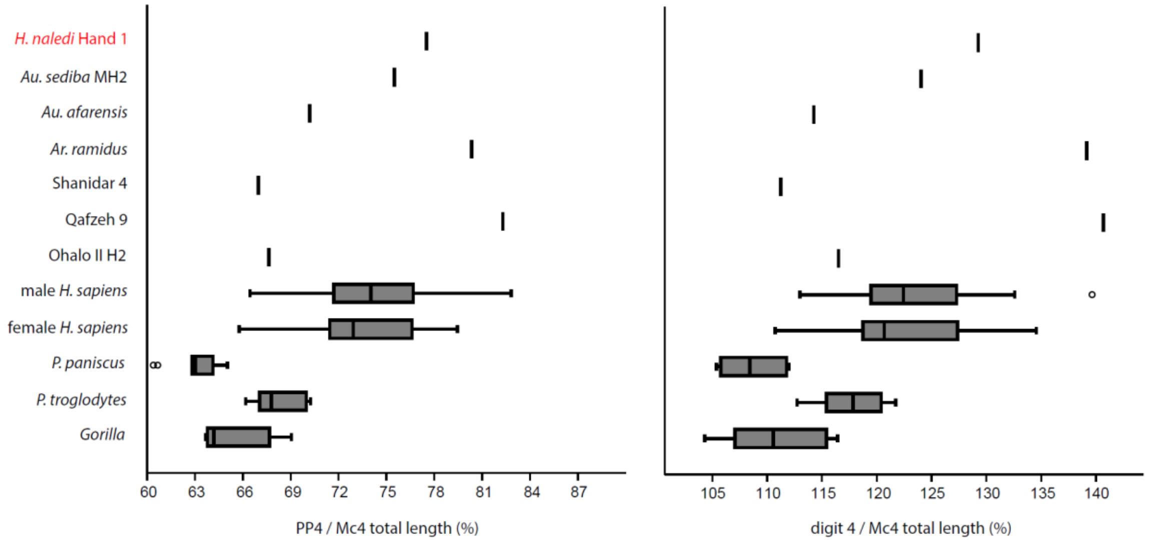

Supplementary Figure 7. *H. naledi* Hand 1 finger-to-palm length proportions. Box-and-whisker plots depicting the total length of the proximal phalanx (PP) or digit length (PP + IP length) divided by total length of the associated metacarpal in (A) ray 2, (B) ray 3, and (D) ray 4 and (C) divided by interarticular length of Mc3 (i.e., not incorporating the styloid process). Kebara 2 is *H. neanderthalensis* and Ohalo II H2 and Qafzeh 9 are early *H. sapiens*. All data collected from original fossils, apart from that of Shanidar 4 derived from Trinkaus (Ref S3). *Au. afarensis* ratios are derived from the composite hand, comprising multiple individuals (Ref S4-5), but hand proportions may be less human-like (Ref S6).

H. naledi finger length of ray two is similar to modern humans while the fingers of ray 3 and ray 4 are long compared with modern humans and most other hominins (apart from *Ar. ramidus*). Hand 1 also has a relatively long PP3 and digit 3 relative to interarticular length of the Mc3 compared to other hominin, but falls within the range of variation of modern humans. However, there is substantial variation in relative PP and digit length across different rays and across different taxa. Early *H. sapiens* Qafzeh 9 is unusual compared to other early and modern *H. sapiens* in having long PPs and digit length for all rays.

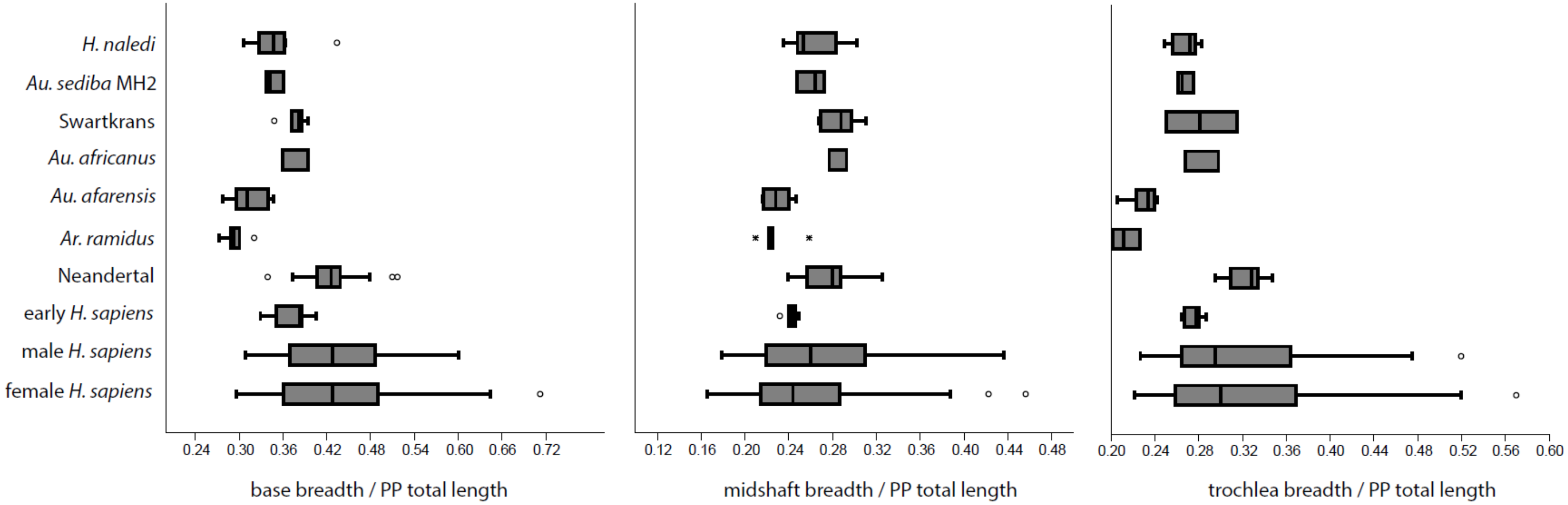

Supplementary Figure 8. Relative robusticity of the *H. naledi* proximal phalanges (PP). Box-and-whisker plots of relative robusticity in all medial proximal phalanges (PP; rays 2-5) in *H. naledi* compared with those of associated or isolated remains of other fossil hominins and modern humans. Depicted is the ratio of maximum mediolateral breadth of the proximal base (left), midshaft (middle) and distal trochlea (right) relative to total length of the proximal phalanx. Fossil sample includes: *H. naledi* (n=11) UW 101-558, -754, -923, -1326, -1327, -1328, -1454, -1460, -1643, -1644, and -1725; *Au. sediba* (n=3) UW 88-108, -120 and -164; Swartkrans specimens attributed to either *Au. robustus* or early *Homo* (n=5) SKX-5018, -15468, -22511+30220, -22741 and -27431; *Au. africanus* (n=2) StW 28 and -293; *Au. afarensis* (n=9) AL 288-1x, AL 333-19, -57, -62, -63, -93, AL 333w-4, AL 1044-1 and AL 444-4; *Ar. ramidus* (n=5) ARA-VP-6/500-030, -022, -069, ARA-VP-7/2H and ARA-VP-6/507; *H. neanderthalensis* (n=13) Shandiar 3, 4, 5 and 6 and Kebara 2; early *H. sapiens* (n=6) Qafzeh 8 and 9. All data derived from original fossils, except for AL288-1x, for which data are derived from Bush et al.[12].

H. naledi proximal phalanges are most similar in overall robusticity to other australopiths, apart from *Au. afarensis*, and modern and early *H. sapiens*. *Ar. ramidus* and *Au. afarensis* are generally less robust because their PP length is longer than later hominins. *Au. africanus*, Swartkrans specimens and particularly Neandertals tend to be slightly more robust than *H. naledi* and *H. sapiens*.

Supplementary Table 1. *H. naledi* sample of hand bones (n=149) from Dinaledi chamber of Rising Star cave system. All bones from the right hand associated with Hand 1 were found in articulation. Associations of bones with Hands 2-5 are more tentative, based on similarity in size and morphology and/or developmental stage. Mc, metacarpal; PP, proximal phalanx; IP, intermediate phalanx; DP, distal phalanx.

Specimen #	Association	Element
**Paratype Hand 1 (adult)**		
U.W. 101-033	antimere to 101-1319	proximal 3/4 Mc3L
U.W. 101-418B	antimere to 101-1732	complete left lunate
U.W. 101-1308		compete IP4R
U.W. 101-1309		complete Mc5R
U.W. 101-1310		complete IP3R
U.W. 101-1311		complete IP2R
U.W. 101-1318		complete Mc4R, excl. part of head
U.W. 101-1319	antimere to 101-033	complete Mc3R
U.W. 101-1320		complete Mc2R
U.W. 101-1321		complete Mc1R
U.W. 101-1325		complete IP5R
U.W. 101-1326		complete PP4R
U.W. 101-1327		complete PP3R
U.W. 101-1328		complete PP2R
U.W. 101-1329		complete DP2R
U.W. 101-1351		complete DP1R
U.W. 101-1464	antimere to 101-1327	complete PP3L, excluding trochlea
U.W. 101-1721		PP1R
U.W. 101-1722		DP3R
U.W. 101-1723		DP4R
U.W. 101-1724		DP5R
U.W. 101-1725		PP5R
U.W. 101-1726		complete right scaphoid
U.W. 101-1727		complete right triquetrum
U.W. 101-1728		complete right trapezoid
U.W. 101-1729		complete right hamate
U.W. 101-1730		complete right capitate
U.W. 101-1731		complete right trapezium
U.W. 101-1732	antimere to 101-418B	complete right lunate
**Additional hand bones**		
**Hand 2 (adult)**		
U.W. 101-713	antimere to 101-1640	partial left hamate
U.W. 101-807	antimere to 101-1639	complete left scaphoid
U.W. 101-1282	antimere of 101-1641	complete Mc1L
U.W. 101-1454	antimere to 101-1645	complete PP5L
U.W. 101-1466		proximal 3/4 of Mc5L
U.W. 101-1639	antimere to 101-807	complete right scaphoid

U.W. 101-1640	antimere to 101-713	complete right hamate
U.W. 101-1641	antimere of 101-1282	complete Mc1R
U.W. 101-1642		complete PP1, pathological
U.W. 101-1643		complete PP3, probably L
U.W. 101-1644		complete PP4, probably R
U.W. 101-1645	antimere to 101-1454	complete PP5R, excluding base
U.W. 101-1646		complete IP3
U.W. 101-1647		complete IP4
U.W. 101-1648		complete IP5
U.W. 101-1649		complete non-pollical DP
U.W. 101-1650		shaft of probable Mc2R
U.W. 101-1651+1628		complete Mc3R
U.W. 101-1653		metacarpal shaft fragment
U.W. 101-1655		phalanx trochlea
U.W. 101-1656		scaphoid fragment
**Hand 3 (adult)**		
U.W. 101-270		complete Mc1R
U.W. 101-916		complete left trapezium
U.W. 101-930	antimere to 101-1385	complete left capitate
U.W. 101-1385	antimere to 101-930	complete right capitate
**Hand 4 (immature)**		
U.W. 101-559		complete Mc3L, excluding head
U.W. 101-517+721		complete Mc3R
**Hand 5 (immature)**		
U.W. 101-1029		metacarpal shaft fragment
U.W. 101-1271		metacarpal shaft fragment
U.W. 101-1536		metacarpal shaft fragment
U.W. 101-1633		metacarpal shaft fragment
U.W. 101-1635		PP shaft with partial trochlea
U.W. 101-1636		metacarpal shaft fragment
U.W. 101-1654		metacarpal shaft fragment
U.W. 101-1664		distal 1/2 of IP
**Isolated hand bones**		
U.W. 101-007		complete Mc1L
U.W. 101-036		metacarpal shaft fragment
U.W. 101-120		partial PP1L base
U.W. 101-168		PP shaft
U.W. 101-175		complete PP, excluding base
U.W. 101-178		complete IP (IP5?)
U.W. 101-381		complete IP
U.W. 101-400		distal half of Mc2L
U.W. 101-401		shaft of Mc1L
U.W. 101-418A		metacarpal shaft fragment
U.W. 101-428		complete PP1L
U.W. 101-512		proximal half of Mc2R
U.W. 101-537		metacarpal fragment

U.W. 101-554	complete proximal phalanx, excluding base
U.W. 101-558	complete PP (PP3 or PP4?)
U.W. 101-603	complete IP, excluding trochlea
U.W. 101-604	complete non-pollical DP
U.W. 101-665	complete IP
U.W. 101-678	phalanx shaft fragment
U.W. 101-720	proximal PP1
U.W. 101-754	complete PP (PP2 or PP4?)
U.W. 101-757	distal half of Mc2L
U.W. 101-777	complete IP (IP5?)
U.W. 101-868	metapoidal shaft fragment
U.W. 101-913	complete PP, excl. base (PP3 or PP4?)
U.W. 101-917	complete Mc1L
U.W. 101-923	complete PP, eroded trochlea
U.W. 101-924	complete IP (IP3 or IP4?)
U.W. 101-982	IP shaft fragment
U.W. 101-1025	complete PP, excl. proximal facet
U.W. 101-1027	complete IP (IP3 or IP4?)
U.W. 101-1055	complete PP1L
U.W. 101-1225	metacarpal shaft fragment
U.W. 101-1232	metacarpal shaft fragment (Mc2L?)
U.W. 101-1247+1630	PP (PP5?)
U.W. 101-1264	metacarpal shaft fragment (Mc3?)
U.W. 101-1265	metacarpal shaft fragment
U.W. 101-1298	proximal half of Mc2L
U.W. 101-1348	PP
U.W. 101-1365	metacarpal shaft fragment
U.W. 101-1379	complete IP, excluding base
U.W. 101-1380	PP shaft
U.W. 101-1424	complete PP, excluding base (PP3?)
U.W. 101-1440	complete IP, with eroded trochlea (IP5?)
U.W. 101-1453	complete DP1R
U.W. 101-1455	complete IP (IP3 or IP4?)
U.W. 101-1460	complete PP (PP2 or PP4?)
U.W. 101-1462	distal 1/4 of Mc2L
U.W. 101-1474	proximal 1/2 of Mc2R
U.W. 101-1478	complete PP, excluding trochlea (PP5?)
U.W. 101-1479	complete IP
U.W. 101-1516	eroded PP shaft, possibly immature
U.W. 101-1527	non-pollical DP
U.W. 101-1539	sagittal half of PP
U.W. 101-1541	metacarpal shaft fragment
U.W. 101-1545	partial right trapezoid
U.W. 101-1546	complete right lunate
U.W. 101-1552	PP epiphysis
U.W. 101-1580	partial right trapezium

U.W. 101-1581	complete right trapezoid
U.W. 101-1582	complete non-pollical DP
U.W. 101-1583	metacarpal head
U.W. 101-1590	complete non-pollical DP
U.W. 101-1607	complete non-pollical DP
U.W. 101-1619	complete IP, excluding base
U.W. 101-1620	PP shaft fragment
U.W. 101-1624	proximal fragment of right scaphoid
U.W. 101-1631	metacarpal shaft fragment
U.W. 101-1632	metacarpal shaft fragment
U.W. 101-1634	metacarpal shaft fragment
U.W. 101-1637	IP shaft fragment
U.W. 101-1652	metacarpal shaft fragment
U.W. 101-1658	metacarpal head
U.W. 101-1660	complete IP (IP2 or IP4?)
U.W. 101-1702	triquetrum
U.W. 101-1703	non-pollical DP
U.W. 101-1704	complete IP
U.W. 101-1705	complete IP, excluding trochlea
U.W. 101-1706	complete IP
U.W. 101-1708	PP fragment with trochlea
U.W. 101-1709	distal end of PP
U.W. 101-1715	immature metacarpal head
U.W. 101-1718	non-pollical DP
U.W. 101-1748	metacarpal shaft fragment
U.W. 101-1749	proximal Mc3R shaft
U.W. 101-1750	metapoidal head frag

Supplementary Table 2. Relative robusticity of *H. naledi* the (A) pollical (DP1) and (B) non-pollical (DP2-5) distal phalanges. All data derived directly from fossils unless otherwise indicated. Estimated values are indicated by parentheses.

(A)

Relative size of DP1 apical tuft
(length of DP1/ML breadth or DP height of apical tuft)

Taxon (specimen)	ML breadth	DP height
Hand 1 (UW 101-1351)	1.39	3.45
H. naledi (UW 101-1453)	1.57	3.64
Ar. ramidus (ARA-VP 6/500-049)	3.40	5.03
Au. afarensis (AL333-159)	2.38	3.28
Au. africanus (StW 294)	1.80	3.73
Au. sediba (UW 88-124)	[1.84]	3.36
Au. robustus (TM1517k)	1.59	3.17
*Au. robustus/*early *Homo* (SKX 5016)	1.75	5.05
H. habilis (OH7)	1.65	4.20
H. neanderthalensis (Kebara 2)[1]	2.06	5.75
H. neanderthalensis (range of *n*=9)[2]	1.68-1.94	4.64-6.24
early *H. sapiens* (Qafzeh 9)[1]	2.24	6.50
modern *H. sapiens* (range)[2]	1.79-2.84	4.37-8.13

[1]Mean value of left and right DP1s
[2]Data derived from Smith (Ref S7), including Kiik-Koba 1, Shanidar 3, 4, 5,6, Krapina 1, 2, 3, 4

(B)

Relative size of non-pollical distal phalanx apical tuft
(length of DP/ML breadth or DP height of apical tuft)

Taxon (specimen)	Ray	ML breadth	DP height
Hand 1 (range of n=4)	DP2-5	1.95-2.30	3.54-3.92
H. naledi isolated (range of n=2)	DP2-5	1.91-2.51	3.42-406
Au. afarensis (AL 333w-11)	?	3.02	4.53
Au. afarensis (AL 333w-50)	?	2.56	3.73
Au. robustus (TM1517o)	?	2.22	4.33
H. habilis (OH7, range of n=2)	DP2-5	2.22-2.36	4.32-4.96
H. neanderthalensis (Kebara 2, range of n=4)	DP2-5	2.25-2.88	4.08-6.35
H. neanderthalensis (Amud 1, range of n=2)	DP2-5	1.94-1.96	4.21-5.02
H. neanderthalensis (Shanidar 4)[1]	DP4?	1.69	4.70
H. neanderthalensis (Shanidar 3)[1]	DP5	2.29	4.89
early *H. sapiens* (Qafzeh 9, range of n=4)	DP2-5	2.22-3.16	4.92-5.73
early *H. sapiens* (Qafzeh 8)	DP2-4?	2.73	4.99

[1]Data from Trinkaus (Ref S3)

Supplementary Table 3. Results from canonical variates analyses of the *H. naledi* wrist bones. Correlations between each (A) scaphoid, trapezium and trapezoid and (B) capitate and hamate variable and canonical axis. See also Figure 5.

(A)

Variable		CAN1	CAN2
**Scaphoid**			
Angle between			
radius	trapezium-trapezoid	-0.08	0.04
capitate	radius	0.02	0.07
capitate	trapezium-trapezoid	0.19	-0.09
Relative surface area			
radius		0.06	0.07
capitate		0.08	0.31
trapezium-trapezoid		0.16	0.04
nonarticular		-0.12	-0.19
**Trapezium**			
Angle between			
1st metacarpal	scaphoid	-0.17	0.11
1st metacarpal	trapezoid	0.11	-0.10
scaphoid	trapezoid	0.05	-0.10
1st metacarpal	2nd metacarpal	0.22	-0.04
2nd metacarpal	scaphoid	-0.13	-0.10
2nd metacarpal	trapezoid	-0.16	-0.09
Relative surface area			
1st metacarpal		0.22	0.19
2nd metacarpal		-0.01	0.01
scaphoid		0.21	0.06
trapezoid		0.11	0.38
Curvature			
radioulnar		0.00	0.03
dorsovolar		0.15	0.06
**Trapezoid**			
Angle between			
lateral 2nd metacarpal	medial 2nd metacarpal	0.01	-0.14
lateral 2nd metacarpal	scaphoid	-0.13	0.00
lateral 2nd metacarpal	trapezium	-0.09	0.15
medial 2nd metacarpal	scaphoid	-0.32	0.25
medial 2nd metacarpal	trapezium	-0.12	0.02
scaphoid	trapezium	0.09	-0.18
Relative surface area			
nonarticular		0.07	-0.47
lateral 2nd metacarpal		0.03	0.18
medial 2nd metacarpal		-0.18	-0.03
scaphoid		-0.29	0.06
trapezium		0.02	0.40

(B)

Variable		CAN1	CAN2
**Capitate**			
Angle between			
hamate	2nd metacarpal	-0.21	0.12
hamate	3rd metacarpal	0.21	0.09
2nd metacarpal	3rd metacarpal	-0.37	0.16
hamate	scaphoid-lunate	-0.13	0.04
2nd metacarpal	scaphoid-lunate	0.33	-0.21
3rd metacarpal	scaphoid-lunate	-0.02	-0.02
Relative surface area			
hamate		0.03	0.17
2nd metacarpal		-0.16	0.29
3rd metacarpal		0.22	-0.24
nonarticular		-0.05	-0.10
scaphoid-lunate		0.14	0.01
**Hamate**			
Angle between			
capitate	4th metacarpal	0.04	-0.08
capitate	5th metacarpal	0.06	-0.13
capitate	triquetrum	-0.06	-0.19
5th metacarpal	triquetrum	-0.01	0.32
4th metacarpal	5th metacarpal	0.00	-0.02
4th metacarpal	triquetrum	0.00	0.28
Relative surface area			
capitate		-0.05	0.26
4th metacarpal		0.27	0.08
triquetrum		-0.03	0.17
5th metacarpal		-0.14	-0.46
Other			
hamulus length index		0.07	-0.20
hamulus angle		0.39	0.16
hamulus cross section index		-0.28	-0.05
5th metacarpal-4th metacarpal index		-0.25	-0.28

Supplementary Table 4. Proximal and intermediate manual phalanges used in the phalangeal curvature analyses. The 1st polynomial coefficient (A) from the polynomial function (y=Ax2+BX+C) for each specimen and sample means are provide. The first coefficient (A) expresses the nature and degree of the longitudinal curvature whereas the second (B) and third (C) reflect aspects of the orientation of that curve with respect to the rest of the element (i.e. element rotation, element position in 2-dimensional space). Only the first coefficient (A) was used in phalangeal curvature analyses. See Supplementary Text for further details on the high-resolution polynomial curve fitting methodology.

Proximal specimen #	1st polynomial coefficient	Intermediate specimen #	1st polynomial coefficient
UW 101-1635	0.057524	UW 101-381	0.101164
UW 101-1326	0.124927	UW 101-665	0.045624
UW 101-1327	0.084024	UW 101-777	0.110766
UW 101-1328	0.084672	UW 101-924	0.097098
UW 101-1348	0.081354	UW 101-1027	0.089341
UW 101-1380	0.133318	UW 101-1308	0.083512
UW 101-1424	0.09052	UW 101-1310	0.11319
UW 101-1454	0.05361	UW 101-1325	0.136254
UW 101-1460	0.072335	UW 101-1455	0.116046
UW 101-1643	0.073648	UW 101-1479	0.116046
UW 101-1644	0.095462	UW 101-1518	0.146949
		UW 101-1646	0.119265
		UW 101-1647	0.126878
		UW 101-1648	0.079093
**Sample Mean**	**0.08649036**	**Sample Mean**	**0.105802**

Supplementary Note 1: Morphological descriptions (including raw data) of *H. naledi* Hand 1 hand bones

CARPALS

UW101-1726 right scaphoid

Preservation This specimen is complete and well-preserved, apart from erosion of the cortex at the tip of the tubercle.

Morphology This scaphoid measures an estimated 19.1 mm in its maximum dimension (roughly ML), 12.7 mm in DP height and 8.4 mm in PD length. The radial facet is continuously convex in both the DP and ML dimensions and measures 11.5 in DP height and 13.6 ML breadth. The capitate facet is shallow and oval-shaped, measuring 10.3 mm in DP height 12.1 mm ML breadth. It is "closed" along the distal border due to a large trapezoid facet. The lunate facet is palmarly-positioned and oriented medially, but is not well-defined, measuring an estimated 6.1 mm in DP height and 6.8 mm PD length. The tubercle is short and palmarly-oriented, and its base robust. In dorsodistal view, the trapezium-trapezoid facet is 8.8 mm ML breadth and 15.6 mm in PD length. It is mildly convex ML and extends onto the scaphoid tubercle.

UW101-1732 right lunate

Preservation This specimen is complete and well-preserved, apart from cortex missing from the distal tip of the palmar beak of the capitate facet and non-articular palmar surface of lunate body.

Morphology This specimen is the antimere to UW 101-418B left lunate. UW101-1732 measures 13.6 mm in PD length, 14.0 mm in DP height and 13.4 mm in ML breadth. The capitate facet measures 11.9 mm in DP height and is ML narrow (7.8 mm). There is no separate articulation for the hamate. The scaphoid facet is poorly defined and measures an estimated 7.6 mm in DP height and 2.7 mm in PD length. The scaphoid facet can be easily seen in distal view, oriented equally laterally and distally. The triquetrum facet measures 8.3 mm in DP height and 7.0 mm in PD length and is mildly convex in both dimensions. The radial facet is broad ML (13.5 mm) and DP (12.4 mm) and dominates the proximal surface.

UW 101-418B left lunate

Preservation This specimen is complete and well-preserved apart from a fragment of cortex missing from dorsal half of the lunate facet and the non-articular palmar surface of lunate body. There is a overall porosity of the cortex on the radial facet.

Morphology The specimen is the antimere to UW 101-1732 right lunate and its overall morphology is identical to that described for UW 101-1732, apart from a slightly more DP convex triquetrum facet. This bone measures 14.4 mm in PD length, 13.7 mm in DP height and 11.6 mm in ML breadth. The capitate facet measures 12.0 mm in DP height and 7.6 mm in ML breadth. The scaphoid and triquetrum facets measure an estimated 9.0 mm and 7.9 mm in DP height, respectively, and 4.4 mm and 7.4 mm in PD length, respectively. The radial facet is 12.4 in DP height and 11.4 ML breadth.

UW 101-1727 right triquetrum

Preservation This specimen is complete and well-preserved, apart from cortex missing from the dorsolateral edge of the hamate facet.

Morphology This triquetrum measures 13.6 mm in ML breadth, 9.9 mm in DP height and 7.2 mm in PD length. The lunate facet measures 7.4 mm in DP height and 6.2 mm in PD length and is mildly concave in both directions, matching the convexity of the lunate's triquetrum facet. . The hamate facet is generally triangular-shaped, measuring 12.4 mm in ML breadth and 7.7 mm in DP height. The lateral half of the hamate facet is flat or mildly concave, while the medial half is strongly convex and oriented equally distally and dorsally. This morphology matches the more dorsally-place triquetrum facet on the hamate. The hamate facet wraps around onto the distal side of the bone at its most medial extent. This portion articulates with a slightly proximally-facing portion of the distal end of the triquetrum facet on the hamate. In palmar view, the lateral half of the triquetrum is nonarticular, convex both ML and PD, and a deep sulcus is not present. The medial half is dominated by the pisiform facet, which is small, oval-shaped and well-defined. It measures 7.6 mm in ML breadth and 5.9 mm in PD length. The pisiform facet is oriented primarily palmarly, but also slightly medially, and is strongly concave ML and generally flat PD. The medial non-articular side is deeply excavated.

UW 101-1731 right trapezium

Preservation This specimen is complete, but much of the cortical surface is eroded, exposing trabecular bone. The palmar, distal and dorsal surfaces have exposed trabeculae, which obscures the extent of the Mc2 articulation. However, the remaining morphology is not obscured.

Morphology This bone measures 19.6 mm in PD length and 13.2 mm in DP height. The Mc1 facet is small relative to the size of the bone and measures an estimated 10.2 mm in PD length and 8.0 mm in ML breadth. The curvature of the ML concavity is relatively flatter than the strong curvature of the DP convexity. The tubercle is large and is extended primarily palmarly, but also slightly proximolaterally. The groove for the flexor carpi radialis tendon is deep. The trapezoid and scaphoid articulations are generally flat and small relative to the overall size of the bone, together measuring 12.2 mm in PD length. The trapezoid facet measures 7.1 mm in PD length and 6.4 mm in DP height. The scaphoid facet measures 7.5 mm in ML breadth. The Mc2 articulation is obscured due to the eroded cortex.

UW 101-1728 right trapezoid

Preservation This specimen is complete and well-preserved, apart from cortex eroded from the dorsal tip of the Mc2 facet exposing trabeculae.

Morphology This trapezoid is boot-shaped and measures 14.4 mm in its maximum dimension (DP height). The non-articular palmar surface is expanded and measures 8.0 mm in ML breadth. The non-articular dorsal surface measures 12.5 mm in ML breadth and 10.7 mm in PD length. The trapezium facet measures 11.5 mm in DP height and 7.1 in PD length. The Mc2 facet measures 10.9 mm in DP height and 9.9 mm in ML breadth, and its medial portion is slightly larger and more DP curved than the lateral portion. The capitate facet is roughly square-shaped and dorsally positioned.

UW 101-1730 right capitate

Preservation This specimen is complete and well-preserved. The cortex is eroded from the palmar surface of the proximal facet, the distal tip of the non-articular palmar surface and dorsal edge of the Mc3 facet.

Morphology This is a small capitate, measuring 19.0 mm in maximum PD length, 14.2 mm in DP height and 12.0 mm in ML breadth. The head measures 10.0 mm in ML breadth and an estimated 9.7 mm in DP height. The scaphoid and lunate articular areas of the proximal facet extend distally to a similar extent and a dorsal ridge at the distal edge is absent. In lateral view, the scaphoid facet blends with a single, dorsally-placed trapezoid facet. A separate palmar trapezoid facet is absent. The trapezoid facet is rectangular-shaped, measuring 3.1 mm in DP height and 4.2 in PD length, and abuts a single, large articular area for the Mc2. The Mc2 facet is oriented equally laterally and distally and is mildly concave DP. It extends almost the complete DP height of the lateral side of the capitate body, measuring 9.8 mm, and is 6.0 mm in PD length. UW101-1324 shows limited waisting of capitate "neck", measuring 8.9 mm in ML breadth. The hamate facet on the medial side measures 13.4 mm in PD length and 7.0 mm in DP height. It does not extend the full length of the medial side of the hamate body, but instead there is a 2.7 mm PD long non-articular area at its distal end. The proximal half of the hamate facet is concave, oriented slightly distally compared with the relatively flat distal half of the facet, consistent with the corresponding morphology on the hamate. The distal Mc3 facet is concave palmarly and in its dorsomedial corner. It measures 11.9 in DP height and 10.6 mm and 6.2 mm in ML breadth at the dorsal and palmar regions, respectively.

UW101-1729 right hamate
Preservation This specimen is generally complete and well-preserved. Trabeculae are exposed at the distodorsal edge of the capitate facet, distodorsal edge of triquetrum facet, and dorsal edge of Mc5 facet. There is erosion of cortex at the tip of the hamulus. The preserved morphology of the hamulus (in medial view, the palmar portion at the end of the hamulus is beginning to flare) suggests that only the cortex is missing and not a large portion of the hamulus itself.
Morphology This specimen measures 19.0mm in maximum (including hamulus) preserved PD length (estimated 20.0 mm complete) and 16.8 mm (estimated 17.4 mm complete) in DP height. The hamate body (excluding the hamulus) measures 16.5 mm in PD length, 10.5 mm in DP height and 13.5 in ML breadth. The hamulus extends distally beyond the hamate body and is PD long and ML narrow. The Mc4 and Mc5 facets measure 9.2 mm and 7.5 mm in DP height and 7.8 mm and 6.5 mm in ML breadth, respectively. Thus, the Mc4 facet is slightly larger than that for the Mc5. The Mc5 facet is mildly saddle-shaped; is strongly curved DP and mildly curved ML and extends slightly onto the base of the hamulus. In dorsal view, the Mc5 facet is oriented equally medially and distally relative to the Mc4 facet. The capitate facet is mildly convex PD and measures 8.5 mm in DP height and 13.3 mm in PD length. It does not extend to the distal border of the hamate body, but instead there is a 2.7 mm gap of non-articular surface, which is reflected in the offset carpometacarpal articulations of the Mc3-capitate and Mc4-hamate. The triquetrum facet measures 6.8 mm in DP height and 12.9 mm in PD length. It is dorsally placed on the hamate, such that there is non-articular surface at the proximal end, palmar to the facet rather than the articular surface extending the entire DP height of proximal hamate. The triquetrum facet is proximally and dorsally oriented at the proximal half, and proximally-palmarly oriented at the distal half, creating the typical concavo-convex triquetrum facet morphology.

METACARPALS

UW 101-1321 right first metacarpal

Preservation This specimen is complete and generally well-preserved. There is cortex missing from the lateral and medial edges of the palmar articular surface of the head and from palmar and lateral surface of proximal shaft, just distal to the trapezium facet.

Morphology This bone measures 37.4 mm and 35.8 mm in total and interarticular PD length, respectively. The Mc1 base and proximal shaft are remarkably small compared to a much more robust midshaft and distal half of the bone. The base and proximal shaft measure 9.6 mm and 6.6. mm in ML breadth and 10.2 mm and 8.6 mm in DP height, respectively. The trapezium facet is also small, measuring 8.3 mm in ML breadth and 6.8 mm in DP height. In comparison, the midshaft and proximal shaft measure 9.5 mm and 11.4 mm in ML breadth, and 8.1 mm and 9.7 mm in DP height. The head measures 11.1 in maximum ML breadth and 12.6 in DP height. In palmar view, the shaft appears "pinched", created by a crest running along the sagittal midline with strongly sloping medial and lateral walls that end in flaring crests for the intrinsic thenar muscles. On the lateral side of the shaft, a well-developed flange for the opponens pollicis tendon that begins roughly 12 mm from the proximal end of the Mc1 and measures 12.3 mm in PD length. The flange for the dorsal interosseous tendon is also distally positioned and measures 11.0 mm in PD length. The distal articular facet is strongly asymmetric with a more palmarly projecting, but less proximally extended, lateral portion compared with the medial portion. UW101-1321 does not have prominent "beak" at the sagittal midline of the head's palmar surface.

UW 101-1320 right second metacarpal

Preservation This specimen is complete apart from palmar and lateral portion of the proximal base and a large fragment missing from the sagittal midline of the dorsal surface of the distal shaft. The cortex is porous on the palmar-lateral surface of the distal articulation and the cortex is eroded at the most proximal extent of the dorsodistal articular surface.

Morphology This Mc2 measures 53.7 mm and 50.9 mm in total and interarticular PD length, respectively. Much of the proximal base is missing, but cortex is preserved along the tip of the trapezoid facet (lateral) and capitate facet so the total length of the bone can be quantified. The articular facets on the proximomedial surface are not well-preserved; articular surface is preserved (6.0 mm in DP height) along the dorsal half of the medial surface for the capitate and Mc3, but it is not possible to distinguish between the two facets or if there is a single or double Mc3 articulation. The articular surface is continuous with the small portion of the trapezoid facet that is preserved on the proximolateral surface of the proximal end. The dorsal surface of the base as two flat protuberances divided by a deep sulcus. The distal portion of the shaft is ML flat and this flatness continues proximally along the shaft, tapering ML until it reaches the lateral protuberance on the dorsal surface of the base. The shaft is robust, measuring 7.3 mm in ML breadth and 6.7 mm in DP height at midshaft, and has a prominent crest running that sagittal midline for the attachment of the palmar interossesous muscles. The lateral side of the distal half of the shaft also flares slightly. There is no noticeable torsion to the shaft. The distal head measures 10.6 mm in maximum ML breadth and 11.3 mm in DP height. In palmar view, the distal articular surface is strongly asymmetrical, with the lateral portion of the articular surface extending much further proximally than the medial portion.

UW 101-1319 right third metacarpal
Preservation This specimen is complete and well-preserved, apart from the erosion of cortex from the dorsal surface of distal articulation and the proximal edge of medial and dorsal surface of the base.
Morphology This specimen measures 49.0 mm and 48.6 mm in total and interarticular length, respectively. The shaft is robust, measuring 6.7 mm in ML breadth and 6.5 mm in DP height, and there is slight crest running along the sagittal midline of the palmar surface, ending at the medial side of the shaft-base junction, suggesting well-developed palmar interosseous muscles. The proximal base measures an estimated 12.2 mm in ML breadth and 11.6 mm in DP height. The dorsolateral corner of the base extends more proximally than the remainder of the base. This is the region of the styloid process in modern humans, though the morphology of UW101-1319 is best described as a slight extension rather than a styloid process. In proximal view, the base is triangular in shape and is dominated by the capitate facet. The capitate facet measures 9.5 mm and 4.1 mm in dorsal and palmar ML breadth, respectively and is largely mildly convex DP, except for a concave dorsolateral corner. There is a single articulation for the Mc2, which can be clearly seen in in palmar view due to its palmar and lateral orientation. The Mc2 facet measures 4.8 mm in PD length and 11.0 mm in DP height. In medial view, there is a single, small, dorsally-placed articulation for the Mc4, measuring an estimated 4.2 mm in PD length and 4.1 mm in DP height. This facet is flat and primarily medially-oriented but also slightly palmarly-oriented. The Mc4 facet is distally-placed on the proximal base, such that in articulation, the Mc4 carpometacarpal articulation is offset distally from the Mc3. This articular configuration is consistent with the morphology of the capitate and hamate as well. The head is strongly asymmetrical towards the lateral side and measures 9.9 in maximum ML breadth and 11.3 in DP height.

UW 101-033 left third metacarpal
Preservation This specimen preserves a partial proximal epiphysis and ¾ of the shaft. A large fragment is missing from the mediodorsal corner of the proximal epiphysis such that the full extent of the capitate is not preserved. Cortex is eroded from the non-articular palmar surface of the base, exposing trabeculae. A piece of the cortex is missing from the dorsomedial non-articular surface, but trabeculae are not exposed.
Morphology This left Mc3 is the likely antimere to 101-1319 right Mc3. The preserved morphology is identical to that described for UW101-1319. This bone measures 36.5 mm in total preserved PD length. DP height of the base is estimated at 12.0 mm. The midshaft measures 6.6 mm in both ML breadth and DP height.

UW 101-1318 right fourth metacarpal
Preservation This specimen is complete apart from a large portion of the lateral and dorsal portions of the distal epiphysis. The cortex is eroded from lateral border of the hamate facet (though re-articulation with hamate shows that full extent of hamate facet is retained).
Morphology This specimen is short and gracile relative to the other metacarpals. It measures 42.7 mm and 42.4 mm in total and interarticular length, respectively. The midshaft measures 5.2 mm in ML breadth and 6.5 mm in DP height. Unlike the other metacarpals of Hand 1, the shaft walls do not flare laterally and a slight crest can be palpated along only the proximal half of the palmar shaft surface. The proximal base is small and square-shaped, measuring 9.2 mm in ML breadth and 9.4 mm in DP height. The hamate facet dominates the proximal surface and is generally flat, apart from a mildly convex dorsomedial corner. The hamate facet measures 7.4 mm in ML breadth and 5.3 mm in DP height and its palmar portion is slightly dorsally oriented such that it can be seen in dorsal view. Laterally, there is a single, dorsally-positioned Mc3 facet measuring 3.8 in PD length and 3.7 in DP height. Medially, the

Mc5 facet is DP concave such that the dorsal portion is slightly palmarly-oriented. This facet measures 7.6 mm in PD length and 4.9 mm in DP height. From the preserved portion, the head appears to be slightly medially rotated relative to the base.

UW 101-1309 right fifth metacarpal

Preservation This bone is complete and well-preserved. The distal articulation has erosion of the cortex on its palmar surface and a circular fragment missing from its dorsal surface.
Morphology This Mc5 measures 40.7 mm and 39.5 mm in total and interarticular length, respectively. The midshaft measures 6.6 mm in ML breadth and 6.1 in DP height. There is well-developed attachment for the opponens digiti minimi tendon along the medial side of the distal shaft, beginning 21.4 mm from the proximal end and measuring 6.3 mm in PD length. The medial tubercle at the base for attachment of flexor carpi ulnaris tendon is moderately developed. The base measures 10.6 mm in ML breadth and 7.9 mm in DP height. The hamate facet measures 6.6 in maximum ML breadth and 6.0 in DP height. It is strongly DP convex and narrow, extending onto the palmar surface of the Mc5 base, and mildly ML concave creating a saddle joint, corresponding to the saddle-shaped Mc5 facet on the hamate. The Mc4 facet is roughly rectangular in shape, mildly DP curved and oriented equally laterally and dorsally, and slightly proximally. This facet measures 4.8 mm in PL length and 8.1 mm in DP height. Medially, there is a smooth but non-articular region, extending from the proximal edge of the base to the dorsal junction of the base and shaft. The head is strongly asymmetrical (laterally rotated) and measures 9.5 mm in maximum ML breadth and 10.6 mm in DP height.

PROXIMAL PHALANGES

UW101-1721 right pollical proximal phalanx

Preservation This specimen is complete apart from a small fragment missing from the palmar surface of the lateral trochlea. The cortex is eroded on the lateropalmar surface of the proximal base.
Morphology This specimen is 24.5 mm and 23.6 mm in total and interarticular PD length, respectively. The base measures 11.8 mm in ML breadth and 9.4 mm in DP height and is asymmetric, with the lateral portion extending more proximally than the medial portion. The basal tubercles are distinct, laterally for the attachment of flexor pollicis brevis and abductor pollicis brevis tendons, and medially for the adductor pollicis tendon. The proximal articular surface is relatively round, measuring 9.2 mm in ML breadth and 8.4 mm in DP height. The shaft is robust relative to the length of the bone, measuring 7.5 mm in ML breadth and 5.4 in DP height. The palmar surface of the shaft is ML flat and dorsal surface is mildly convex. Although a portion of the lateral trochlea is missing, the trochlea appears ML broad (10.9 mm) relative to the breadth of the shaft and base. The missing portion of the lateral trochlea obscures an assessment of its asymmetry.

UW 101-1328 right second proximal phalanx

Preservation This specimen is complete and well-preserved. There is slight erosion of the cortex along the lateral-palmar border of the lateral trochlea.
Morphology This specimen measures in 32.2 mm in total PD length. The dorsal shaft is strongly convex PD, especially at the distal 1/3 of the phalanx. The palmar surface is generally ML flat and slightly concave PD and lacks convexity at the sagittal midline. The palmar shaft is generally featureless, with poorly-developed flexor sheath ridges that do not extend palmarly above the remaining surface of the shaft. Overall the shaft is not robust relative to its length. The midshaft measures 8.5 mm in ML breadth and 5.0 mm in DP

height. The proximal articulation is oval-shaped, measuring 10.8 mm in ML breadth and 8.4 mm in DP height, and strongly concave both ML and DP. The proximal base, measuring 11.7 mm in ML breadth and 9.3 mm in DP height, also is not particularly robust and the basal tubercles are present, but not distinct. The base is asymmetric, with the lateral basal tubercle being more prominent and the lateral portion extending more proximally than the medial portion. Distally, the medial trochlea is slightly more distally extended than the lateral trochlea. Together, the asymmetry of the base and trochlea are typical for right second phalanx. The trochlea measures 9.1 mm in ML breadth and 5.7 mm in DP height. There is a slight concavity just proximal to the trochlea on the palmar surface of the shaft.

UW 101-1327 right third proximal phalanx

Preservation This specimen is complete and well-preserved apart from a small fragment missing from the medial border of the trochlea.

Morphology The third proximal phalanx shows the same general morphology as that described in UW 101-1328 from the second ray. Subtle differences include slightly more well-developed flexor sheath ridges, such that there is a slight indentation along the proximal half of the medial border of the palmar surface. However, the ridges still do not extend palmarly above the remainder of the shaft surface. The palmar tubercles and distal trochlea are more symmetrical than in the other *H. naledi* proximal phalanges. This specimen 35.9 mm in total PD length. The base and proximal facet measure 12.0 mm and 8.9 mm in ML breadth and 10.5 mm and 8.0 mm in DP height, respectively. The midshaft and distal trochlea measure 8.9 mm and 9.2 mm in ML breadth and 6.0 mm and 6.3 mm in DP height, respectively.

UW 101-1326 right fourth proximal phalanx

Preservation This specimen is complete apart from a fragment missing from the dorsomedial boundary between the shaft and proximal articulation. The cortex is eroded from the palmar surface of the medial trochlea.

Morphology This specimen shows same general morphology as the other proximal phalanges of Hand 1. The proximal base is slightly asymmetrical with the lateral part of the proximal facet extending further proximally then the medial side. There is a very slight convexity to the lateral side of the shaft for the flexor sheath ridge and only the very distal portion extends slightly beyond the remainder of the palmar shaft. The lateral trochlea extends slightly further distally than the medial side, consistent with typical asymmetry for right PP4. This PP4 measures 33.1 mm in PD length. The base measures 11.5 mm in ML breadth and an estimated 9.1 mm in DP height. The proximal facet measures 10.4 mm in ML breadth. The midshaft and distal trochlea measure 8.9 mm and 9.0 mm in ML breadth and 5.3 mm and 5.8 mm in DP height, respectively.

UW 101-1725 right fifth proximal phalanx

Preservation This specimen preserves the complete proximal ¾ of the shaft but is missing the trochlea. The cortex is eroded from the proximomedial border of the dorsal surface.

Morphology This specimen is similar in overall morphology to that described for the other proximal phalanges associated with Hand 1. The proximal end is asymmetrical, with the medial side extending further proximally than the lateral side. In dorsal view, the proximal facet is asymmetrically placed such that is shifted medially. This specimen 25.5 mm in preserved PD length and is estimated to be approximately 26.5 mm if the trochlea were fully preserved. The base and proximal facet measure 9.3 mm and 7.6 mm in ML breadth and 8.4 mm and 8.3 mm in DP height, respectively. The midshaft is 8.0 mm in ML breadth and 4.7 mm in DP height.

INTERMEDIATE PHALANGES

UW 101-1311 right second intermediate phalanx

Preservation This specimen is complete apart from a small hole on the proximomedial corner of the palmar surface of the shaft, a large fragment of cortex missing from the distal half of the dorsal shaft and a smaller fragment missing from the laterodorsal surface at the proximal edge.

Morphology This specimen is 17.8 mm in total PD length. The proximal base is ML broadest portion of the bone, and the walls of the shaft taper distally toward the trochlea. The shaft is ML narrowest just before the trochlea and the trochlea is nearly as ML expanded as the proximal base. The dorsal shaft is continuously DP convex, and is particularly curved at distal end (although not as pronounced as in the proximal phalanges). The proximal border of the dorsal surface is slightly flaring. The palmar shaft morphology has two convex semilunar areas in the proximal half of the shaft. The region in between the semilunar areas and the entirety of the distal half of the palmar shaft is ML flat, apart from a slight concavity at the sagittal midline just proximal to the trochlea. A well-developed palmar median bar is not present. The trochlea is more ML expanded than all but the most proximal region of the shaft. The base and proximal facet measure 9.2 mm and 8.5 mm in ML breadth and 6.8 mm and 5.1 mm in DP height, respectively. The midshaft and distal trochlea measure 5.7 mm and 7.4 mm in ML breadth and 4.5 mm and 4.3 mm in DP height, respectively.

UW 101-1310 right third intermediate phalanx

Preservation This specimen is complete apart from a small fragment missing from the centre of the proximal border of the palmar surface. The cortex is slightly eroded at the proximal border of the dorsal surface.

Morphology This specimen is 22.6 mm in total PD length. The morphology is identical to that described for UW 101-1311 second intermediate phalanx, except that the proximal border of the dorsal surface is slightly more flaring. The bicondylar proximal articulation is fully preserved and each is oval-shaped, being ML broader (9.4 mm together) than they are DP tall (5.9 mm). The base is 10.5 mm in ML breadth and 7.1 mm in DP height. The midshaft and distal trochlea measure 7.6 mm and 8.1 mm in ML breadth and 4.9 mm and 5.0 mm in DP height, respectively.

UW 101-1308 right fourth intermediate phalanx

Preservation This specimen is complete apart from a small fragment missing from the centre of the proximal border of the palmar surface.

Morphology This specimen is 22.1 mm in total PD length. Its morphology is identical to that described for UW101-1310, apart from a mildly ML concave, rather than flat, surface between the convex semi-lunar areas of the palmar surface and there is a slight palmar extension to the lateral border of the shaft, likely for the attachment of the flexor digitorum superficialis tendon. There is also less dorsal flaring at the proximal border than in UW101-1310. The base and proximal facet measure 10.6 mm and 9.1 mm in ML breadth and 7.1 mm and 6.3 mm in DP height, respectively. The midshaft and distal trochlea measure 7.6 mm and 7.9 mm in ML breadth and 4.4 mm and 4.4 mm in DP height, respectively.

UW 101-1325 right fifth intermediate phalanx

Preservation This specimen is complete and well-preserved.

Morphology This specimen is 15.6 mm in total PD length. Its morphology is identical to that described for the other intermediate phalanges of Hand 1, with a few subtle differences. The lateral facet of the proximal articulation is DP taller than the medial facet. The semilunar

convexities of the palmar shaft are ML narrower such that they appear more as ridges, separated by a more pronounced concavity than is seen in the other intermediate phalanges. The distal half of the palmar shaft remains ML flat and there is no median bar as is typical of the other specimens. The base and proximal facet measure 8.9 mm and 8.0 mm in ML breadth and 6.4 mm and 5.5 mm in DP height, respectively. The midshaft and distal trochlea measure 6.0 mm and 7.0 mm in ML breadth and 3.6 mm and 3.7 mm in DP height, respectively.

DISTAL PHALANGES

UW 101-1351 right pollical distal phalanx
Preservation This specimen is complete and well-preserved apart from small fragments missing from the lateral and dorsal borders of the proximal base, and the erosion of the cortex along the proximal border of the base and the lateral edge of the apical tuft. The palmar surface of the apical tuft also appears slightly eroded.
Morphology This pollical distal phalanx is remarkably ML broad relative to its length. It measures 15.2 mm in total PD length. The base and midshaft measure an estimated 12.4 mm and 9.9 mm in ML breadth and an estimated 6.0 mm and 4.8 mm in DP height. The base is not well-preserved but it is clear that the proximal articular surface is DP short (estimated 4.6 mm). The dorsal surface of the bone is convex in both the ML and DP dimensions. The palmar surface has a deep, large and circular proximal palmar fossa measuring 8.9 mm in ML breadth and 6.2 mm in DP height. The distal border of the palmar fossa has a roughened, palmarly-extended ridge for the attachment of the flexor pollicis longus (FPL) tendon. The ungual fossa between the FPL attachment and the apical tuft is slightly concave (though this may be accentuated by slight erosion of the palmar surface as well) and relatively short in PD length. The apical tuft is very ML expanded (10.9 mm) but DP narrow (4.4 mm), giving an overall broad and flat appearance to the bone. The medial tip of the apical tufts proximal edge (lateral side is eroded) flares medially and proximally as an ungual spine.

UW 101-1329 right second distal phalanx
Preservation This specimen is complete and well-preserved apart from erosion of cortex along the proximal border of the dorsal surface.
Morphology This specimen is 12.7 mm in total PD length. The base is robust, measuring 7.5 mm in ML breadth and 4.9 mm in DP height. The palmar surface of the proximal half of the shaft has a deep and somewhat circular excavation with a prominent distal edge for the attachment of the flexor digitorum profundus tendon. In sagittal view, the dorsal shaft is DP mildly concave along the proximal half and mildly convex along the distal half. The midshaft, measuring 4.3 mm in ML breadth and 3.9 mm in DP height, is ML narrow compared with the broader base and, particularly, the very broad apical tuft, but DP tall. The apical tuft measures 6.5 mm in ML breadth and 3.3 mm in DP height. The medial proximal tip of the apical tuft expand laterally and slightly proximally as a ungula spine, but similar morphology is not seen on the lateral side.

UW 101-1722 right third distal phalanx
Preservation This specimen is missing most of its medial side, broken in the sagittal plane just medial to the sagittal midline. However the full length of the lateral ¾ of the bone is well-preserved.
Morphology This specimen measures 14.0 mm in preserved PD length and an estimated 14.4 mm in total PD length. The preserved morphology is identical to that described for UW101-1329 apart from a deep palmar fossa. The base measures 5.3 mm in DP height. The midshaft

and apical tuft measure 4.5 and an estimated 6.9 mm in ML breadth and 3.8 mm and 3.8 mm in DP height, respectively.

UW101-1723 right fourth distal phalanx

Preservation This specimen is complete and perfectly preserved, apart from slight erosion of the cortex on the lateral-proximal border of the palmar surface.

Morphology This specimen is 14.1 mm in total PD length. The morphology is identical to that described for UW 101-1329, except that the ML breadth of the base and apical tuft are even more pronounced and both the medial and lateral proximal tips of the apical tuft project slightly proximally and palmarly as ungual spines. The base measures 9.3 mm in ML breadth and 5.2 mm in DP height. The midshaft and apical tuft measure 4.4 and 7.0 mm in ML breadth and 3.5 mm and 3.6 mm in DP height, respectively.

UW 101-1724 right fifth distal phalanx

Preservation This specimen is complete and perfectly preserved apart from cortical erosion along the proximal border of the palmar surface.

Morphology This specimen is 12.4 mm in total PD length. The morphology is identical to that described for UW 101-1329, except that the palmar fossa is not as deep and the apical tuft is not as ML expanded, as would be expect for a DP5. The proximal border of the dorsal surface is more dorsally flaring than in the other non-pollical phalanges of Hand 1. The base measures 7.0 mm in ML breadth and 5.1 mm in DP height. The midshaft and apical tuft measure 3.5 and 5.4 mm in ML breadth and 3.5 mm and 3.5 mm in DP height, respectively.

Supplementary References:

1. Orr, C.M. *et al*. New wrist bones of *Homo floresiensis* from Liang Bua (Flores, Indonesia). *J. Hum. Evol.* **64**, 109-129 (2013).
2. Niewoehner, W.A., Weaver, A.H. & Trinkaus, E. Neandertal capitate-metacarpal articular morphology. *Am. J. Phys. Anthropol.* 103, 219-233 (1997).
3. Trinkaus, E. *The Shanidar Neandertals* (Academic Press, London, 1983).
4. Markze, M.W. Joint functions and grips of the *Australopithecus afarensis* hand, with special reference to the region of the capitate. *J. Hum. Evol.* **12**, 197 (1983).
5. Alba, D.M., Moyà-Solà, S. & Köhler, M. Morphological affinities of the *Australopithecus afarensis* hand on the basis of manual proportions and relative thumb length *J. Hum. Evol.* **44**, 225 (2003).
6. Rolian, C. & Gordon, A.D. Reassessing manual proportions in *Australopithecus afarensis. Am. J. Phys. Anthropol*. **152**, 393-406 (2014).
7. Smith, S. L. Shape variation of the human pollical distal phalanx and metacarpal. *Am. J. Phys. Anthropol.* **113**, 329-348 (2000).
